# A Novel Mutation of the *NARROW LEAF 1* Gene Adversely Affects Plant Architecture in Rice (*Oryza sativa* L.)

**DOI:** 10.3390/ijms21218106

**Published:** 2020-10-30

**Authors:** Prasanta K. Subudhi, Richard S. Garcia, Sapphire Coronejo, Teresa B. De Leon

**Affiliations:** 1 School of Plant, Environmental, and Soil Sciences, Louisiana State University Agricultural Center, Baton Rouge, LA 70803, USA; Rgarcia@agcenter.lsu.edu (R.S.G.); scoronejo@agcenter.lsu.edu (S.C.); 2 California Cooperative Rice Research Foundation, Inc., Biggs, CA 95917, USA; tdeleon@crrf.org

**Keywords:** brassinosteroids, chimeric transcript, chlorophyll content, gibberellin, photosynthesis, pleiotropy, *Oryza sativa*, RNA-Seq

## Abstract

Plant architecture is critical for enhancing the adaptability and productivity of crop plants. Mutants with an altered plant architecture allow researchers to elucidate the genetic network and the underlying mechanisms. In this study, we characterized a novel *nal1* rice mutant with short height, small panicle, and narrow and thick deep green leaves that was identified from a cross between a rice cultivar and a weedy rice accession. Bulked segregant analysis coupled with genome re-sequencing and cosegregation analysis revealed that the overall mutant phenotype was caused by a 1395-bp deletion spanning over the last two exons including the transcriptional end site of the *nal1* gene. This deletion resulted in chimeric transcripts involving *nal1* and the adjacent gene, which were validated by a reference-guided assembly of transcripts followed by PCR amplification. A comparative transcriptome analysis of the mutant and the wild-type rice revealed 263 differentially expressed genes involved in cell division, cell expansion, photosynthesis, reproduction, and gibberellin (GA) and brassinosteroids (BR) signaling pathways, suggesting the important regulatory role of *nal1.* Our study indicated that *nal1* controls plant architecture through the regulation of genes involved in the photosynthetic apparatus, cell cycle, and GA and BR signaling pathways.

## 1. Introduction

Plant architecture is a three-dimensional arrangement of plant organs [1]. It plays an important role in enhancing agronomically important traits and the adaptability of plants. Although plant architecture to some extent is influenced by environmental factors such as light, temperature, plant density, and humidity, it is inherently complex due to the involvement of multiple molecular regulatory mechanisms and hormone signaling pathways influencing several component traits. Elucidation of the molecular mechanisms underlying rice plant architecture provides a foundation for designing crop plants that can efficiently harvest light energy, absorb nutrients, resist diseases and pests, and accommodate high-density planting for improved crop yield and quality.

Phenotypic mutants (both spontaneous and induced) are important resources for the functional characterization of relevant genes using both forward and reverse genetics approaches [2]. A large number of mutants have been generated using physical mutagens, chemical mutagens, and transposable elements for systematic functional genomics studies [3]. The availability of genome sequences, and genetic and physical maps of crop plants is accelerating the cloning and molecular genetic characterization of traits relevant for agronomic and evolutionary studies. Mutants that are defective in plant architecture allow researchers to elucidate the genetic network and the underlying mechanisms that can aid in manipulating plant ideotype for crop improvement [1,4].

Rice is an ideal model for basic and applied research and is as attractive as Arabidopsis due to its small genome size, well-developed transformation system, availability of abundant germplasm, and genomic resources. The rice plant displays a variety of architectures that are determined by a constellation of traits such as plant height, tiller number, tiller angle, leaf length, leaf width, leaf angle, and panicle and grain characteristics, which are crucial for improved productivity. Genes responsible for many of these component traits have been cloned: plant height [5], internode length [6], leaf morphology [7], leaf length [8,9], and panicle characteristics [10,11].

Optimal leaf characteristics are crucial for improving rice yield. Natural variations in leaf size have been reported among rice accessions [12]. A dwarf narrow leaf mutant (*nal1*) had a defective vascular system with decreased number of longitudinal veins due to defective polar auxin transport [13]. *Nal1* (LOC_Os04g52479) encodes a putative trypsin-like serine and cysteine protease. It was the focus of several subsequent investigations, which demonstrated its potential in enhancing rice productivity [14,15,16]. These studies have shown that some major quantitative trait loci (QTL) allelic to *Nal1* pleiotropically affect yield-enhancing traits. The *GREEN FOR PHOTOSYNTHESIS (GPS)* QTL in rice was identical to *Nal1*, in which increased photosynthesis was conferred by a partial loss-of-function allele [15]. The superior *Nal1* allele increased mesophyll cell number, leaf thickness, and photosynthesis rate with no undesirable phenotype such as dwarf stature, as seen in *nal1* mutants. A major QTL (*qLSCHL4*) for flag leaf shape and chlorophyll content was allelic to the *Nal1* locus [16]. Their overexpression and near-isogenic lines analyses demonstrated the utility of *Nal1* in enhancing chlorophyll content, flag leaf size, and plant type. The gene, *SPIKELET NUMBER (SPIKE)*, identified in a tropical *japonica* landrace, was allelic to *Nal1* that enhanced yield in *indica* cultivars by increasing spikelet number, leaf size, root system, and number of vascular bundles [14]. A major QTL for flag leaf width (*qFLW*) colocalized with this locus and alternate splicing was cited as the cause for the functional difference between both alleles [17]. Regulation of the source–sink relationship by the *Nal1* locus has been reported [18].

Analysis of *Nal1* mutants revealed that they control leaf width and plant height by regulating cell division at the beginning of leaf development [19]. They regulate both leaf and adventitious root development at the transcriptional level [20]. Taguchi-Shiobara et al. [21] identified a significant QTL for flag leaf width corresponding to *Nal1*. Based on the examination of the role of *nal1, nal2, nal3*, and *nal7* mutants in the regulation of leaf width and vein number, it was concluded that these genes play different roles in controlling narrow leaf phenotypes [22]. In a study involving nonallelic narrow leaf mutants, *Nal2* and *Nal3* [9], the expression levels of auxin-transport and leaf development-related genes were altered. An RNA-Seq experiment conducted using leaves of a 40 day-old *nal1* mutant and wild-type revealed that a large number of genes involved in cell wall formation were downregulated [23]. Another study showed that *Nal1* interacts with the *FRIZZY PANICLE (FZP)* gene and promotes its degradation. The downregulation of the *FZP* gene or upregulation of *Nal1* was suggested to increase the grain yield by increasing the number of secondary branches and grain number [24].

Despite the role of *Nal1* in a wide array of plant development processes, as described in the above studies, further investigation is needed to understand the molecular basis of pleiotropic effects of this important gene on agronomically important traits. The discovery of a deletion in the *nal1* locus and its deleterious impact on plant architecture led us to hypothesize that the mutation in this locus alters the regulation of genes involved in several developmental and hormonal signaling pathways, leading to undesirable morphological changes. In this study, we characterized a spontaneous mutant with altered plant architectural attributes such as short height, small panicles, increased chlorophyll content (SPAD values), and narrow and thick deep green leaves identified from an advanced backcross population from the cross between a cultivated rice and a weedy rice accession. Bulked segregant analysis coupled with whole-genome re-sequencing and cosegregation analysis in two mapping populations revealed that the overall phenotype was controlled by the mutant *nal1* gene with recessive inheritance. The drastic changes in plant architecture was caused by a deletion spanning over the last two exons, 3′ untranslated region (UTR), and a part of the intergenic region between *Nal1* and the adjacent gene resulting in formation of chimeric transcripts. Although *Nal1* natural variants were reported to impart beneficial effects leading to increased productivity and enhanced photosynthetic activity, detrimental effects of a *nal1* mutant were demonstrated in this study.

## 2. Results

### 2.1. Phenotypic Characterization of the W149 Mutant

**Figure 1:**
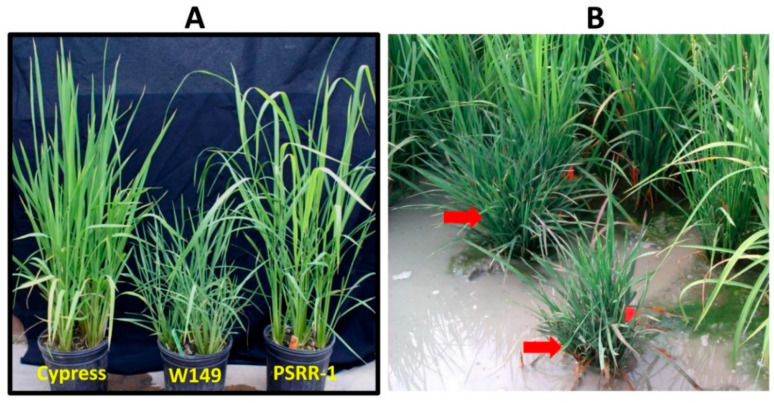
(**A**) Overall morphology of Cypress (Left), W149 (middle), and PSRR-1 (right); (**B**) segregation of mutant and normal plants in field condition. The mutant plants are marked by red arrows.

W149 had a shorter plant height with more tillers, shorter and narrower leaves, and higher chlorophyll content than Cypress (recurrent parent) (Table 1; Figure 1). W149 plants were 40% shorter than Cypress. The panicle length and all internodes of W149 were significantly shorter than Cypress, except for internode II. The panicle length had the most contribution to the total culm length of both Cypress and W149. In Cypress, the contribution of internodes toward the total culm length was I > III > V > IV > II, whereas the internode order was I > II > III > V > IV in W149 (Figure S1). There was a significant reduction in the culm area in W149 compared with Cypress. However, the stem vascular system did not show noticeable abnormality (Figure S2). The leaf vascular systems of Cypress and W149 showed no significant differences in the number of major/large veins (LV), but the reduction in minor/small veins (SV) between LV was apparent in W149. There were five to seven SV between LV in Cypress leaves compared to two to five in W149. In addition, the midribs of Cypress leaves were more prominent than W149. The interveinal distance (IVD) was shorter in W149 compared to Cypress. The shortening of IVD in W149, along with a reduced number of SV, contributed to the narrowing of the leaves. Another distinguishing feature of the W149 mutant was its leaf thickness (Table 1). Compared to Cypress, W149 leaves were 55% thicker due to more bundle sheath extensions and larger bundle sheath cells (BSC). The vascular bundle (VB) area was larger in W149 leaves than in Cypress leaves. However, no difference was found in the mesophyll cell (MC) area between Cypress and W149. In addition, W149 leaves were deep green with a higher SPAD reading than Cypress leaves.

### 2.2. Bulked Segregant Analysis and Whole-Genome Sequencing Reveals Involvement of Nal1 Locus

Morphological evaluation of 1200 F_2_ plants from the W149 × Cypress cross showed 279 plants with mutant phenotype, which fit into the 3 normal:1 mutant ratio. To confirm that the W149 with altered plant architecture is a mutant rather than an introgression line, a W149 × PSRR-1 F_2_ population was generated. Morphological evaluation of 191 F_2_ plants showed 51 mutant phenotypes with monogenic segregation, an observation consistent with the earlier result. Both hybrids from W149 × Cypress and W149 × PSRR-1 cross-combinations also showed normal morphology, suggesting involvement of a recessive gene controlling the overall mutant phenotype. Bulked segregant analysis (BSA) using the normal and mutant plants from the W149 × PSRR-1 F_2_ population showed several markers with clear linkage on chromosome 4. A partial linkage map generated using polymorphic markers indicated that the putative mutant locus was located between RM255 and RM3836 (Figure S3).

**Figure 2:**
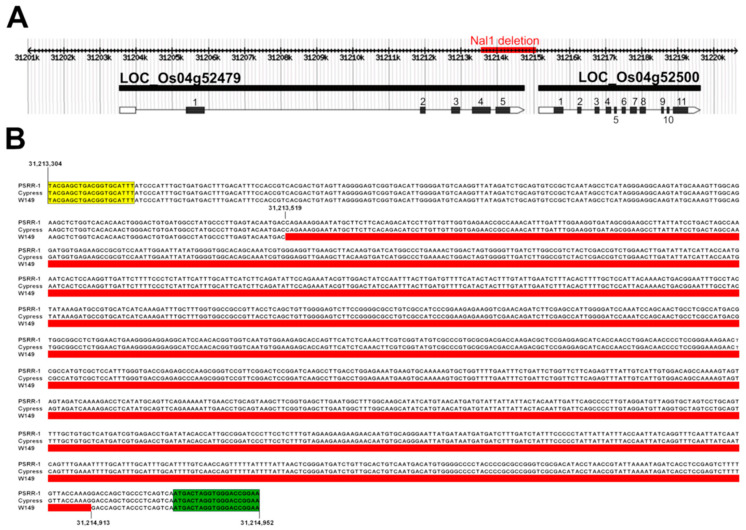
Location of the 1395-bp deletion in the *nal1* locus (LOC_Os04g52479) in W149 genome. (**A**) The deletion encompasses a part of exon 4, exon 5, 3′ untranslated region (3‘UTR), and downstream regions of LOC_Os04g52479, indicated by red bars. (**B**) Sequence alignment of PSRR-1, Cypress, and W149 *Nal1* alleles containing the deletion (highlighted in thick red bar) with genomic coordinates 31,213,519-31,214,913 of chromosome 4. The deletion was verified by Sanger sequencing of PCR fragments. Forward and reverse primers are highlighted with yellow and green boxes, respectively.

Whole-genome sequences of W149, Cypress, and PSRR-1 were assembled, and coverages were 12×, 21×, and 35×, respectively. The counts of the identified variants such as single nucleotide polymorphism (SNPs) and insertions-deletions (InDels) revealed 97.1% Cypress-specific variants and only 2.9% PSRR-specific variants, indicating W149 to be nearly isogenic to Cypress (Table S1). As BSA analysis earlier revealed the presence of the mutant locus between RM255 and RM3836 on chromosome 4, genomic sequences of W149 and Cypress for this region were compared. Although there were no nonsynonymous SNPs or InDels, a large deletion (1395-bp) in the *Nal1* locus (LOC_Os04g52479) was detected in W149. The deletion spanned a part of the exon 4, exon 5, 3‘untranslated region (UTR), and several base pairs of the *Nal1* downstream region (Figure 2). The deletion was absent in both PSRR-1 and Cypress and was confirmed by sequencing of the PCR fragments flanking the deletion. The deleted region was present between the genomic coordinates 31,213,519 and 31,214,913 on chromosome 4.

### 2.3. Genotyping and Phenotyping in Crosses Involving W149

**Figure 3:**
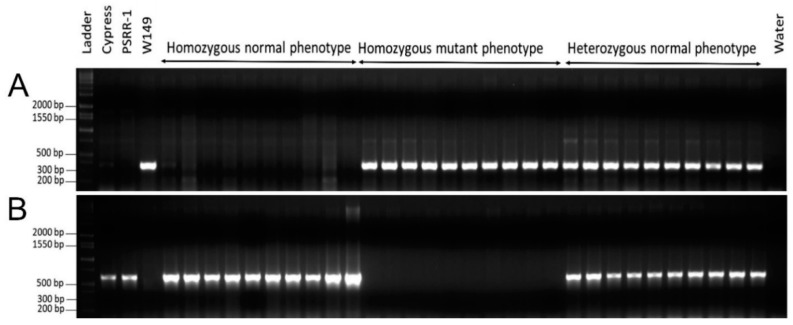
Genotyping of representative F_2_ progenies of the cross W149 × Cypress to distinguish homozygous normal, heterozygous normal, and homozygous mutant plants. (**A**) PCR amplification by the Primer pair 1 (1F and 1R); (**B**) PCR amplification by the Primer pair 3 (2F and 2R).

The populations from W149 × Cypress and W149 × Bengal crosses consisted of 340 and 319 F_2_ plants, respectively. Gross morphological assessment of the F_2_ populations from W149 × Cypress and W149 × Bengal crosses showed 83 and 72 plants with mutant phenotypes, respectively, which fit into a segregation ratio of 3 normal:1 mutant. The genotyping result using the *nal1* gene primer pairs matched with the phenotyping results in both populations, W149 × Cypress (Figure 3) and W149 × Bengal (Figure S4), confirming the *nal1* gene as responsible for the mutant phenotype in W149 and the mutant phenotype as a recessive trait. Few heterozygous F_2_ plants from both crosses, which were confirmed by *nal1* genotyping, were selected and advanced to F_3_ generation. Both phenotyping and genotyping in F_3_ generation further confirmed the cosegregation of the *nal1* deletion mutation with overall mutant phenotype (Tables S2 and S3).

### 2.4. Hormonal Response

**Figure 4:**
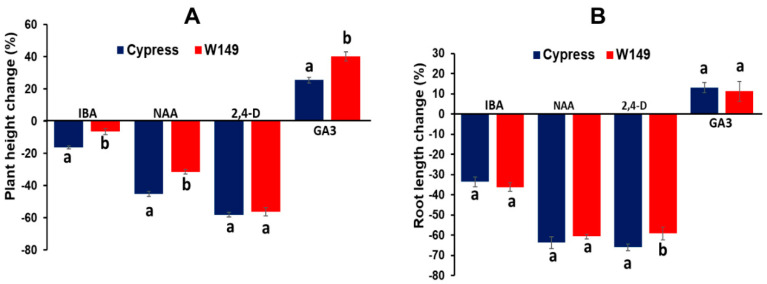
Morphological responses of Cypress and W149 mutant to various plant hormones application. Variations in plant height (**A**) and root length (**B**) after treatment of various phytohormones (0.5 mg/L of nutrient solution) compared to untreated (control) are shown. The hormones are indole-3-butyric acid (IBA), 1-naphthaleneacetic acid (NAA), 2,4-dichlorophenoxyacetic acid (2,4-D), and gibberellic acid (GA3). Means with similar letters indicate no difference in one-way ANOVA analysis (*p* < 0.05; n = 10).

Despite the role of auxins in shoot elongation, there was reduction in plant height and root length in Indole-3-butyric acid (IBA), 1-Naphthaleneacetic acid (NAA), and 2,4-dichlorophenoxyacetic acid (2,4-D) treatments (Figure 4). The reduction in plant height was greater for IBA and NAA treatments in W149 compared with Cypress, and the root length showed the same trend for only 2,4-D treatment. On the contrary, GA3 increased both traits in Cypress and W149. Although there was a positive response to GA application on the plant height and root length of W149, the response to GA3 was significantly greater for plant height in W149 compared with Cypress. The application of GA was able to rescue the W149 by increasing plant height and root length like Cypress.

### 2.5. Comparative Transcriptomics

**Figure 5:**
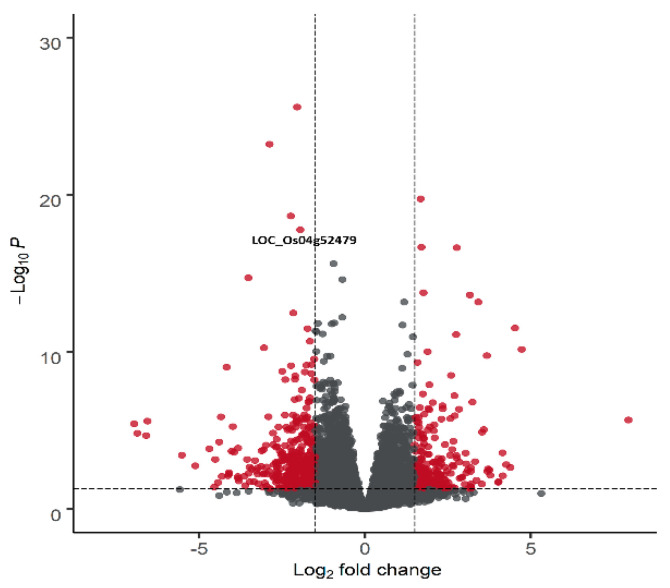
Volcano plot to identify differentially expressed genes in W149 compared with Cypress using DESeq2 R Package. The mean log_2_ fold change is plotted against the −log_10_ Benjamini–Hochberg-adjusted P-values for all expressed genes. X-axis dotted line represents log_2_ fold change threshold (≥1.5 and ≤−1.5) while y-axis dotted line is the adjusted *p*-value cut-off point (<0.05). Red dots represent 330 differentially expressed genes. Gray dots represent genes that did not pass the filtering criteria. Plot was visualized using R package “EnhancedVolcano.”

**Figure 6:**
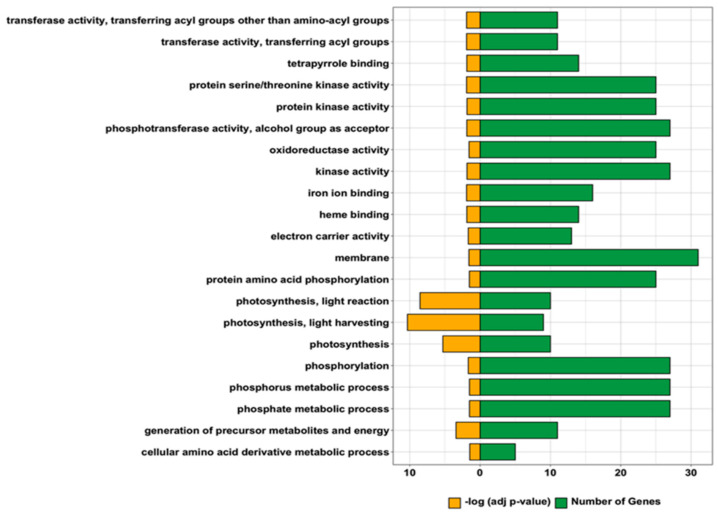
Gene ontology (GO) analysis using agriGO v2.0 reveals enrichment of the differentially expressed genes in W149 compared with Cypress. The significant GO terms are shown on the panel on the left. Yellow bars represent −log (adjusted *p*-values), whereas green bars show the number of genes for each GO term.

We compared the expression profiles of W149 and Cypress to identify the differentially expressed genes (DEGs). An average of 48 and 51 million reads was obtained from Cypress and W149, respectively, with a mapping rate of 95% to the reference genome in both (Table S4). Three hundred and thirty DEGs were identified in W149 compared with Cypress (Figure 5; Table S5). Among these, 122 genes were upregulated and 208 genes were downregulated. From the 330 DEGs, 186 are annotated in the agriGOv2.0 database and 99 DEGs were classified with significant gene ontology (GO) terms. Twenty-one GO terms were significantly enriched in W149. These included 9 GO terms in biological processes (e.g., photosynthesis, phosphorylation, cellular amino acid derivative metabolic process), 11 in molecular function (e.g., protein serine/threonine kinase activity, oxidoreductase activity, transferase activity), and 1 in cellular component (membrane) (Figure 6).

We further filtered the 330 DEGs to identify genes that are putatively involved in the mutant phenotype regulated by the *nal1* locus. As SNPs, InDels, and structural variations (SVs) may alter the gene expression, we examined the sequences of DEGs from the whole-genome sequence data and identified 58 genes including *nal1* under this category (Table S5). The remaining DEGs were checked using the KEGG (Kyoto Encyclopedia of Genes and Genomes) database (www.genome.jp/kegg/kegg1.html) for possible involvement within common network pathways. To illustrate, LOC_Os06g40970 and LOC_Os12g09770 were differentially expressed due to the SNPs and fall in the same KEGG pathway (phenylpropanoid biosynthesis). However, there were DEGs such as LOC_Os03g13210, LOC_Os03g55420, LOC_Os04g59200, and LOC_Os08g02110, which have no genetic mutation but were present in the phenylpropanoid biosynthesis pathway. *LOC_Os06g40970* (DEG with genetic mutation) and LOC_Os03g13210, LOC_Os03g55420, LOC_Os04g59200, and LOC_Os08g02110 (DEGs without genetic mutation) were in the same enzyme class (peroxidases) and phenylpropanoid biosynthesis pathway, which converts p-coumaryl alcohol to p-hydroxy-phenyl lignin. Hence, the differential expression may be due to such interactions. After eliminating such genes, 263 DEGs most likely regulated by *nal1* were finally selected (Table S6). These genes were initially GO-enriched and were manually curated for their biological functions using Phytozome, RAP-DB databases, and available literatures. Grouping of these genes was done according to their biological functions: metabolism (2%), transport (3%), photosynthesis and/or chloroplast-related processes (6%), flowering and seed-related processes (6%), plant growth and development (6%), stress response (18%), and unknown functions (59%) (Figure S5). For quantitative real-time polymerase chain reaction (qRT-PCR) validation, DEGs with biological functions such as photosynthesis and/or chloroplast-related processes, flowering and seed-related processes, and plant growth and development were focused because they reflected the pleiotropy actions of the *nal1* mutation.

The differentially expressed genes (DEGs) involved in plant growth and development were mostly downregulated (Table S6). These genes comprised plant signaling elements, cell growth and expansion, transcription factors, and enzyme proteins. The photosynthesis-related genes were chloroplastic translation proteins, chlorophyll a/b-binding proteins, and chloroplastic enzyme proteins. Most of the flowering and seed-related genes were downregulated, and these were signaling elements, transcription factors, and enzyme proteins. There were no *PIN* or *ARF* genes involved in auxin transport and distribution among the DEGs except an auxin efflux carrier gene (LOC_Os08g41720), which had two SNPs located in the intronic region. On the other hand, we found two DEGs each associated with GA and BR signaling pathways such as LOC_Os01g06210 (gibberellin receptor *GID1L2*), LOC_Os03g41060 (GASR2-Gibberellin-regulated GASA/GAST/Snakin family protein precursor), LOC_Os11g31540 (Brassinosteroid Insensitive 1-associated receptor kinase 1), and LOC_Os02g13900 (brassinazole-resistant 1 [*BZR4*] protein homolog). The downregulation of *GID1L2* may be due to loss of the stop codon and two nonsynonymous SNPs.

### 2.6. Identification and Validation of Chimeric nal1 Transcripts

**Figure 7:**
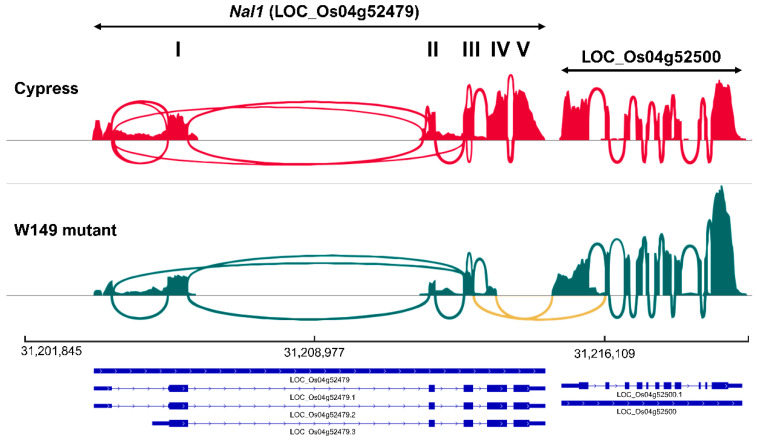
Sashimi plots showing spliced junction (marked yellow) between *Nal1* (LOC_Os04g52479) and LOC_Os04g52500 in W149 compared to Cypress. The height of the bars represents read coverage. The splice junctions are displayed as loops. The exon numbers I through V of *Nal1* are indicated on the top of the panel. The MSU v7.0 gene model with three known transcript variations of *Nal1* is displayed below. The plots were visualized using IGV (v.2.8.4, Broad Institute, Cambridge, MA, USA).

**Figure 8:**
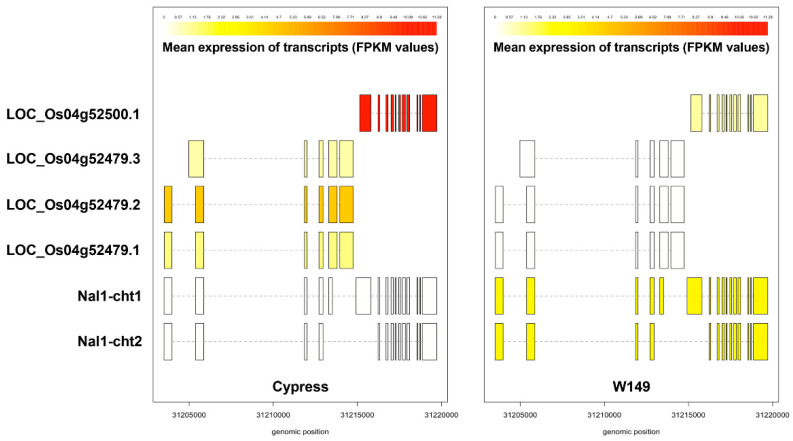
Transcripts identified in W149 and Cypress using Stringtie (v2.1.0). The LOC_Os04g52479.1 and LOC_Os04g52479.2 transcripts are ~2.6 kb in length while the LOC_Os04g52479.3 transcript is ~2.5 kb. The LOC_Os04g52500.1 is located downstream of the *Nal1* gene with ~2.4 kb in length. The Nal1-chimeric transcript 1 (Nal-cht1) arises through splicing of the partial *Nal1*-exon 4 (*Nal1*-ex4) of W149 to the exon 1 of LOC_Os04g52500.1, producing a transcript with a length of ~4.3 kb. The Nal1-cht2 arises when *Nal1*-exon3 (*Nal1*-ex3) is spliced directly to the exon2 of LOC_Os04g52500.1, resulting in a ~3.2 kb transcript. The color shading represents the fragments per kilobase of transcript per million reads (FPKM) values of each transcript produced from Stringtie analysis. The transcripts were visualized using the R package “ballgown.”

The deleted region of nal1 in W149, which included part of the exon 4, exon 5, 3′-UTR, and downstream regions, was confirmed in the RNA-Seq analysis. The adjacent downstream gene LOC_Os04g52500 (lecithine cholesterol acyltransferase) was downregulated in W149 compared with Cypress. While Cypress expressed all known Nal1 transcripts, W149 formed two types of chimeric transcripts with LOC_Os04g52500. In one chimeric transcript (Nal1-Cht2), the exon 3 of LOC_Os04g52479 merged with the exon 2 of the LOC_Os04g52500 and the transcript continued up to the end of LOC_Os04g52500. The second chimeric transcript (Nal1-Cht1) was generated by the fusion of exon 4 of LOC_Os04g52479 with the exon 1 of LOC_Os04g52500 (Figure 7). These chimeric transcripts were identified in all three replicates of W149 RNA-Seq data but not in any replicate of Cypress (Figure S6). Although the reference-guided transcript assembly of RNA-Seq data revealed both chimeric transcripts with similar expression level (Figure 8; Table S7), we could confirm only the Nal1-Cht2 chimeric transcript from the cDNA of W149 by PCR amplification and sequencing (Figure S7). The unsuccessful amplification of the Nal1-Cht1 chimeric transcript could be due to very low abundance for PCR amplification.

### 2.7. qRT-PCR of Differentially Expressed Genes

**Figure 9:**
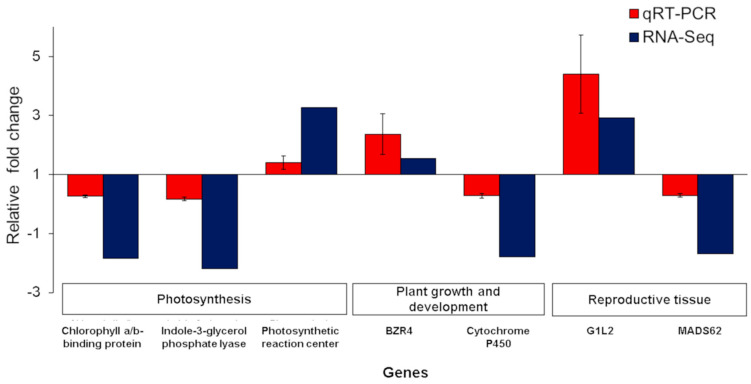
Relative mRNA expression of seven differentially expressed genes in W149 compared with Cypress. Genes plotted are representative of each significant GO term such as photosynthesis, plant growth and development, and reproductive tissue. The y-axis represents the relative mRNA expression value (mean ± standard deviation) from quantitative real-time PCR (qRT-PCR) and RNA-Seq results. Cypress was used as the control and the *EF1α* gene was used as the endogenous control. The genes are chlorophyll a/b-binding protein (LOC_Os01g52240), indole-3-glycerol phosphate lyase (LOC_Os03g58290), photosynthetic reaction center protein (LOC_Os10g39880), brassinazole-resistant 1 protein (BZR4) (LOC_Os02g13900), cytochrome P450 (LOC_Os01g24780), G1 Like Protein 2 (G1L2) (LOC_Os06g46030), and *OsMADS62* (LOC_Os08g38590).

The relative mRNA expression of seven representative differentially expressed genes (three involved in photosynthesis, two each involved in plant growth and development and reproduction) based on RNA-seq analysis was compared in W149 relative to Cypress. Overall, the qRT-PCR results validated the expression pattern of these genes from the RNA-seq analysis, suggesting the reliability of data (Figure 9). Among the photosynthesis-related genes, chlorophyll a/b-binding protein (LOC_Os01g52240) and indole-3-glycerol phosphate lyase (LOC_Os03g58290) showed downregulation while photosynthetic reaction center protein (LOC_Os10g39880) was upregulated in W149 compared with Cypress. In the case of the other two groups of genes related to plant growth and development and reproduction, one gene in each group was upregulated and downregulated. The upregulated genes were *BZR4* (LOC_Os02g13900) and *G1L2* (LOC_Os06g46030), whereas cytochrome P450 (LOC_Os01g24780) and *MADS62* (LOC_Os08g38590) were downregulated.

## 3. Discussion

Since the demonstration of *Nal1* controlling narrow leaf phenotype [13], several studies have highlighted the utility of natural variants of this gene to improve rice productivity due to its favorable pleiotropic effects on many agronomic traits: enhanced photosynthesis, increased flag leaf size, leaf width, panicle length, number of spikelets, regulation of source–sink relationship, and adventitious root development [14,15,16,17,18,20]. Most of these published studies involved normal statured plants with natural *Nal1* variants. However, we characterized a novel *nal1* mutant in this study and compared the nature of this mutation and its phenotypic consequences and the associated mechanisms with previously reported mutants and variants.

### 3.1. Origin and Nature of the Mutation

As the mutant line W149 was identified in an advanced backcross generation involving a weedy rice as the donor, it was initially suspected to be due to introgression of weedy rice genomic segments. However, our mapping experiment followed by PCR sequencing and whole-genome sequence analysis revealed a large deletion at the 3′ end of the *Nal1* gene. Chromosomal rearrangements were known to be triggered due to ‘genomic shock’ during hybridization [25]. The spontaneous mutations, novel chromosomal changes, and retrotransposon activations had been reported in several plant species during hybridization [26,27]. As the weedy rice accession is genetically distant to *japonica* cultivars [28], this mutation most likely spontaneously originated from this distant cross. The absence of any narrow leaved plant in the introgression line population of ‘PSRR-1′ in another rice cultivar ‘Bengal’ background [29] provided further evidence against the origin of this mutant through introgression.

Jiang et al. [19] used two *nal1* mutants to investigate its role in cell division in which *nal1-2* had a deletion of a whole promoter and 404 bp of exon 1 and *nal1-3* mutant had a 6-bp deletion in exon 1. The *nal1* mutant of Qi et al. [13] had 30 bp deletions in the 4th exon. An earlier study grouped the natural variants in rice germplasm into seven categories [21]. The *Nal1* variant *(SPIKE)* had three amino acid substitutions between IR64 (*indica*) and Daringan (*tropical japonica*) [14]. The same three amino acid substitutions differed between narrow leaved Koshihikari (*temperate japonica*) and Takanari *(indica)* with normal leaf width and high photosynthesis [15]. There was an insertion of a 5895-bp retrotransposon in the 2nd exon of Koshihikari and a 30-bp deletion in Takanari. On the other hand, Nipponbare and 93-11 (*indica*), which were used to map QTLs for leaf shape and chlorophyll content, differed in promoter region in addition to the same three nonsynonymous SNPs [16]. The *nal1* allele of D50 (narrow leaf) (*tropical japonica*) used by Chen et al. [17] was the same as IR64 and 93-11. In this study, sequence comparison revealed a 1395-bp deletion in the *nal1* gene in W149 compared to Cypress. Cypress sequence was identical to Nipponbare but differed from PSRR-1 in three nonsynonymous (ns) SNPs.

We demonstrated that due to loss of the stop codon, two different types of chimeric transcripts were generated by fusion of the readthrough partial transcripts of *nal1* (LOC_Os04g52479) and the adjoining LOC_Os04g52500. Preference for the transcript (exon 3-exon 2) was observed, and this differential production of chimeric transcripts was validated by RNA-Seq data. This finding suggests that our mutant is unique and a rare molecular event most likely led to the formation of this chimeric gene. A human fusion gene, KUA-UEV, generated through readthrough transcription and alternative splicing, has been reported [30]. In rice, a chimeric rice gene *GN2* controlling grain number evolved due to insertion of a 1094-bp fragment from LOC-Os02g45150 into the exon 3 of LOC_Os02g56630 from the wild rice *O. rufipogon* [31]. The formation of chimeric genes reported in the rice genome largely involved retrotransposition [32]. As chimeric genes can have both beneficial and detrimental consequences on phenotype due to the evolution of novel functions, this may provide clues for the synthesis of artificial genes in the future [33]. Although several kinds of splice variants for *Nal1* transcripts were reported in both *japonica* and *indica* cultivar [15,16,17], production of chimeric transcripts involving the adjacent gene was not reported earlier, which warrants further investigation to explain the phenotypic consequences of *nal1* based on variation in the expression pattern.

### 3.2. Alteration in Plant Architecture Due to Pleiotropic Action of Nal1

In this study, we demonstrated that *nal1* deletion was correlated to the overall mutant phenotype. Several studies have implicated *nal1* to pleiotropic effects on rice plant architecture, photosynthetic rate, leaf morphology, and grain yield using mutants [13,19,20] and natural variants [14,15,16,17,18,21]. Takai et al. [15] indicated that the partial loss-of-function *nal1* allele in ‘Takanari’ was correlated to increased photosynthetic performance and grain yield with little to no undesirable effect on the plant morphology and physiology compared with ‘Koshihikari’. Compared to the above studies, complete loss-of-function due to a large deletion involving the stop codon in the W149 mutant had a detrimental effect on the plant architecture despite enhanced chlorophyll content, which was consistent with the observation in the *nal1*-mutant of ‘Taichung 65’ and RNAi-*Nal1* transgenic plants [15].

In W149, enhanced cell division in the periclinal orientation and inhibition of cell division in the anticlinal direction were responsible for short stature with thick, narrow, and shortened leaves like an earlier studied mutant [19]. The W149 culm diameter/area and panicle neck reduction were accompanied by a reduction in vascular bundles along the stem periphery. There were variations in height reduction among the mutants used by researchers. In addition, disruption in the stem vascular arrangement of W149 was not evident, contrary to the *nal1* mutant used by Qi et al. (2008) [13]. Such differences in the phenotypes among the *Nal1* mutants suggest that the level of severity and degree of pleiotropy varied among *nal1* mutants and natural variants with different genetic backgrounds.

W149 showed an altered vein patterning due to the reduced number of SVs between two LVs while maintaining normal numbers of LVs. As SVs develop later than the LVs during leaf development [34,35], the limited space between LVs may hinder the formation of SVs. Although variation in polar auxin transport activities has been reported to influence the change in vein patterning like the reduction in SVs observed in *nal1* mutants [13,20], the absence of any auxin transport-related genes among the DEGs indicated a different mechanism controlling the plant architecture in W149. Significant reduction in grain yield in the complete loss-of-function *nal1* mutant W149 was in complete agreement with earlier studies [14,15,24].

### 3.3. Physiological and Molecular Basis of Mutant Phenotype

Due to the pleiotropic nature of the *Nal1*, it has been promoted as a developmental tool to modulate leaf characteristics for enhanced photosynthesis and yield [36]. Compared to 4277 DEGs identified in a *nal1* mutant [23], we identified only 330 DEGs in the transcriptome analysis of the W149 mutant, which could be largely attributed to the near-isogenic nature of the mutant. The nature of mutation, genetic background, and spatial and temporal differences during tissue sample collection may have contributed to such a variation in number of DEGs. Regarding the nature of mutation, Qi et al. [13] used a *nal1* mutant containing a 30-bp deletion, whereas our mutant harbored a large deletion with loss of the stop codon, resulting in a chimeric gene.

Another remarkable phenotype of our *nal1 mutant* was its dark green and thick leaves. About ten DEGs were at least related to photosynthesis and/or chloroplastic activities. These include photosynthetic reaction center protein, Type I chlorophyll a/b-binding protein b (*Lhcb1.1*), and NADPH-dependent oxidoreductase, while other genes involved in chloroplastic protein translation activities were 30s and 50s ribosomal proteins and translation initiation factor IF-1. A chloroplastic precursor, indole-3-glycerol phosphate lyase, was also differentially expressed [37]. Takai et al. [15] concluded that the partial loss-of-function *nal1* allele was responsible for thick leaves and enhanced photosynthesis without any detrimental effect, leading to increased productivity. Although our *nal1* mutant had thick leaves and high chlorophyll content, severely altered plant architecture made it highly unproductive. It is obvious that *nal1* in this study was nonfunctional due to the loss of nuclear localization signal peptides and serine/cysteine protease domains. Therefore, further investigation is needed to explore the function of the chimeric transcripts for understanding the impact of this mutation on rice plant growth and development.

Previous studies showed that *Nal1* was responsible for mutant phenotypes through regulation of polar auxin transport and cell division [13,19,23]. In those studies, the members of the auxin response factor (ARF) gene family and *PIN*-like genes involved in auxin signaling were downregulated. There was only one auxin efflux carrier gene (LOC_Os08g41720), which was downregulated and had two SNPs in the intronic region. It is possible that this gene might be regulated by *nal1*. However, the involvement of this gene could be ruled out because downregulation of this gene in our study had undesirable effects on plant architecture and grain yield, which was contradictory to an earlier study [38], in which reduced expression of the gene had beneficial effects such as increased tiller number, better root system, longer panicles, and enhanced grain yield. Apart from this gene, no ARF gene showed differential expression, suggesting different molecular and physiological mechanisms controlling the mutant phenotype.

Our expression studies also revealed genes involved in cell growth and cell wall synthesis (i.e., *Acyltransferase 19*, *A-expansin*, and *ß-expansin* precursors), leaf and culm development (i.e., *Brittle Culm 7, Homeobox 10, Cell Proliferating Factor 5*), and flowering and seed-related genes (i.e., *Heading Date 3A, Flowering Time Locus 1, Mads Box Gene 62, Receptor-Like Cytoplasmic Kinase 82*). Several cytochrome P450 genes, which are involved in plant growth and developmental processes and stress responses, were downregulated in W149.

### 3.4. Role of Gibberellins (GA) and Brassinosteroid (BR)-Related Genes

Both phytohormones, GA and BR, are known to regulate many physiological and developmental processes in plant [39,40], and therefore, significant crosstalk occurs between the genes involved in signaling and biosynthesis pathways [41,42]. The GA responsiveness of W149 was indicative of disruption in either GA signaling or biosynthetic pathway [41]. Two downregulated genes involved in GA signaling pathways were identified in W149. LOC_Os01g06210 (*GID1L2*) is a soluble gibberellin receptor involved in GA signaling through interaction with a GA signaling repressor *SLR1* [43], whereas LOC_Os03g41060 (GASR2-Gibberellin-regulated GASA/GAST/Snakin family protein precursor) has a role influencing the plant developmental process through their involvement in hormone crosstalk [44]. Based on network analysis in the string database (https://string-db.org/network), this gene may be interacting with many indole-3-acetic acid-amido synthetase genes involved in a mechanism to deal with excess auxin.

Brassinosteroids (BR) are known to regulate the biosynthesis of GA in rice [41,42]. A closer look at the expression profile of plant growth and development-related genes revealed two important genes essential for the brassinosteroid (BR) signaling pathway: LOC_Os11g31540 (Brassinosteroid Insensitive 1-associated receptor kinase 1 [*BAK1*]) and LOC_Os02g13900 (brassinazole-resistant 1 protein homolog [*BZR4*]). *BAK1* plays an important role in mediating the BR signaling pathway regulating plant growth and development [45]. Downregulation of *BAK1* reduced the cell number in leaves, which was consistent with our observation in W149 with respect to gene regulation and mutant phenotype. On the other hand, *BZR4,* which was upregulated in the W149 mutant, is a transcriptional repressor for the target genes encoding key enzymes for BR biosynthesis [46]. In our study, both *BAK1* and *BZR4* most likely affected the levels of BRs and expression of BR-responsive genes. BRs are involved in controlling photomorphogenesis and chloroplast development via direct interactions with BR signaling pathways and key transcriptional regulators [47]. As BRs also play an important role in vascular development [48], the altered expression of BR signaling elements may be responsible for reducing the number of SVs in the W149 mutant. The differential expression of some BR signaling elements (i.e., *BAK1* and *BZR4*) in GA-responsive W149 suggested their involvement in regulating both BR and GA signaling and/or biosynthetic pathways.

## 4. Materials and Methods

### 4.1. Plant Materials and Cultivation

Two rice cultivars (Cypress and Bengal) and a weedy rice accession ‘PSRR-1’ were used for this study. Both rice cultivars were developed by the LSU Agricultural Center [49,50]. ‘PSRR-1’ is a tall medium-grain weedy accession with light green leaves, red pericarp, high seed shattering, and deep seed dormancy. Staggered planting of the above rice genotypes was done to synchronize flowering for crossing. Emasculation was performed by the clipping method, and pollination was done manually the following day. Advanced backcrossed materials were generated by successive backcrossing to the recurrent rice cultivar. The plants were cultivated in semi-controlled greenhouse conditions maintained at 27/21 °C day/night temperature at the greenhouse complex of the Louisiana State University Agricultural Center, Baton Rouge, LA, USA.

### 4.2. Origin of the W149 Mutant

While developing introgression lines of the weedy rice accession ‘PSRR-1’, a mutant plant with an altered plant architecture was identified in the BC_4_F_2_ generation. The mutant plant was genotyped with the polymorphic simple sequence repeat (SSR) markers spread over the rice genome [51]. As the mutant harbored chromosome segments from chromosomes 3, 4, and 12, it was again backcrossed to ‘Cypress’ for one more generation to generate a population of 279 F_2_ plants from which one plant with a homozygous mutant phenotype (named as W149) and single chromosome segment on chromosome 4 was identified in the BC_5_F_2_ generation (Figure S8; Figure 1).

### 4.3. Phenotypic Characterization of W149 Mutant

Comparative phenotyping of Cypress and W149 was done by measuring several quantitative traits such as plant height (PH), tiller number (TN), panicle length (PL), leaf length (LL), leaf width (LW), and leaf greenness or chlorophyll content (SPAD values); the leaf morphological parameters such as leaf vein pattern, leaf thickness (LTH), interveinal distance (IVD), mesophyll cell (MC) area between two veins, vascular bundle (VB) area, bundle sheath cell (BSC), and bundle sheath extension area; and the culm profile such as internode length, culm, and panicle neck size. For the above traits, measurements were taken in the following manner: pH was measured from the base of the plant to the tip of the longest leaf; PL was measured from the start of panicle node to the end of the panicle; LL was measured from the base to tip of the penultimate leaf; LW was measured at the widest portion of the penultimate leaf; and the greenness of leaf was measured thrice at the midportion of the penultimate leaf using a SPAD-502 chlorophyll meter (Spectrum Technologies, Inc., Aurora, IL, USA).

Leaf morphological studies were performed by observing the surface and cross-sections of the leaves. The leaf surface images of fresh leaf samples were taken to determine the vein patterns by counting the major/large veins (LV) and minor/small veins (SV). Leaves were stored in 2% glutaraldehyde (*v/v* of phosphate buffer) at 4 °C for further leaf section analyses. The free-hand section technique was used to view the detailed leaf morphology of the wild-type and mutant. Thin leaf sections were stored in 0.1 M phosphate buffer and transferred to 100% sodium hypochlorite solution for about 1 min to clear the leaf sections. Cleared leaf sections were rinsed with phosphate buffer followed by staining with a droplet of 0.5% toluidine blue for 2 min and washing in distilled water. Leaf sections were viewed under a compound microscope (Fisherbrand^TM^ Micromaster Model CK, Fisher Scientific, Houston, TX, USA) to check the clarity of the leaf sections, and then images were taken using a Leica DM6B microscope (Leica Biosystems, Buffalo Grove, IL, USA). For leaf morphological measurements, images were analyzed using Image J ver. 1.8.0 software and the leaf morphological differences between wild-type and mutant were tested for their significance using Student’s t-test. For the culm profile study, lengths of all internodes were measured and the contribution of each internode to the total plant height was determined. The cross-sections of culm internodes and panicle neck were taken to measure the differences in sizes (area) between the wild-type and the mutant. The procedure for the stem sections and analyses was like the leaf sections mentioned earlier.

### 4.4. Hormonal Response

Morphological responses of Cypress and W149 mutant to various plant hormones such as IBA, NAA, 2,4-dichlorophenoxyacetic acid (2,4-D), and gibberellic acid (GA3) were examined. Rice seeds of both genotypes were sterilized with 1.7% NaClO and germinated on petri-plates under 28 °C for 5 days. Ten germinated plants were transferred to each hydroponics setup without any hormones (control) and with 0.5 mg of each hormone per liter of Yoshida solution. Plants were grown for four weeks and plant height and root length were measured. The change in plant height and root length in each genotype to hormone treatment compared to the control was expressed in percent, and one-way ANOVA was used to determine significant differences in response of different hormone treatments compared to the control.

### 4.5. Mapping Populations for Cosegregation Test and Bulk Segregant Analysis (BSA)

F_2_ populations were developed from three cross-combinations: W149 × PSRR-1, W149 × Cypress, and W149 × Bengal. All three populations were evaluated for overall morphology and the population from the W149 × PSRR-1 was used for BSA [52]. DNA from parents and the mapping populations were extracted from young leaf tissues using the cetyltrimethylammonium bromide (CTAB) method [53]. For mapping of the mutant locus, thirty mutant-type plants and thirty normal-type plants were selected to constitute the mutant-bulk and normal-bulk, respectively. Both mutant-bulk and normal-bulk along with parents W149 and PSRR-1 were screened using 200 simple sequence repeat (SSR) markers distributed over the rice genome. A partial linkage map was constructed using Mapmaker 3.0 for mapping the mutant locus.

### 4.6. Whole-Genome Sequencing (WGS) and Analysis of W149, Cypress, and PSRR-1

The DNA samples from W149, Cypress, and PSRR-1 were sent to the Virginia Bioinformatics Institute (Blacksburg, VA, USA). Genomic libraries were constructed and sequenced using paired-end sequencing on an Illumina HiSeq 2000 platform using standard Illumina protocols [54]. A Burrows–Wheeler Aligner (BWA; MEM-algorithm; version 0.7.8 [55]) was used for the mapping of the high-quality filtered reads against the rice reference genome (Os-Nipponbare-Reference-IRGSP-1.0, downloaded from the Rice Genome Annotation Project (http://rice.plantbiology.msu.edu/cgi-bin/ORF_infopage.cgi)) [56,57]. Structural variants (SV) were called using TIDDIT [58] while genomic variant discovery was done using GATK4 [59]. Additionally, hard filtering was done using VariantFiltration with the basic filtering threshold discussed in the GATK Best Practices documentation. Genome-wide coverage was estimated using SAMtools version 1.9 [55]. SnpEff version 4.3 [60] was used to determine the effects of single nucleotide polymorphisms (SNPs), insertions-deletions (InDels), and structural variants. All bioinformatic analyses were performed using high-performance computing resources available at the Louisiana State University (http://www.hpc.lsu.edu).

### 4.7. Confirmation of the Nal1 Deletion

The primer pair used to confirm the *Nal1* deletion was as follows: Nal-Del-F (5′-TACGAGCTGACGGTGCATTT-′3) and Nal-Del-R (5′-TTCCGGTCCCACCTAGTCAT-′3). For PCR amplifications, a 20 µL PCR cocktail was prepared for each sample containing the following components: 2.0 µL of 10× PCR buffer, 0.4 µL of Taq polymerase, 2.0 µL of 10 µM of each primer, 0.4 µL of 10 mM dNTPs, 1.2 µL of mM MgCl_2_, 3.0 µL of 100 ng/µL DNA, and 9.0 µL distilled water. The following PCR cycle condition was followed: Initial step of 94 °C for 2 min, 35 core cycles (94 °C for 30 s, 55 °C for 20 s, 72 °C for 30 s), and final step of 72 °C for 7 min. About 18 µL of each PCR sample was mixed with 2.0 µL of 10× loading dye before loading and electrophoresed in a 1.5% agarose gel at 100 V for 60 min. The amplification bands with correct size (Cypress:1650 bp and W149: 250 bp) were cut from the gel and purified using a Gel/PCR DNA fragment extraction kit (IBI scientific, Dubuque, IA, USA). Samples were sequenced from both directions via Sanger sequencing at the Genomics Facility of the Louisiana State University.

### 4.8. Phenotyping and Genotyping of Nal1

Phenotyping based on the overall morphology was performed to quantify the number of F_2_ plants having the phenotype like Cypress (recurrent parent) and Bengal (different background) and W149. Each F_2_ plant was scored as either mutant or normal phenotype based on overall morphology, and the Mendelian segregation was tested using the chi-square test.

Co-segregation of the *nal1* allele with the mutant phenotype was determined by genotyping two F_2_ populations using two primer pairs spanning the deleted region of the *nal1* gene:

NalDel-W149-1F (5′-TGCCGCCCCATTATGGATTT-3′),
NalDel-W149-1R (5′-GTACGCCAAACCGGATGAAG-3′),
NalDel-CPRS-2F (5′-GGTGATAGCGGAAGCCTTATT-3′),
and NalDel-CPRS-2R (5′-CGACATACCGACGAAGTTTGA-3′).

The first set of primer (1F and 1R) was used to detect the deletion in the *nal1* gene; Cypress, Bengal, and PSRR-1 alleles had amplifications (608 bp), but there was no amplification for the W149 allele. The second primer set (2F and 2R) was used to identify the heterozygous plants for the *nal1* gene in the mapping populations (Cypress and Bengal: 1774 bp and W149: 376 bp). Two primer sets were used instead of the NalDel_W149 primer set alone to confirm the *nal1* deletion because NalDel_W149 showed some bias in amplifications between two fragments due to a large difference in size (1774 and 376 bp) for heterozygous samples. The design of the nested primers was explained (Figure S9). PCR amplifications and electrophoresis were done in the same way as described earlier. Genotypes were scored and the fitness-to-Mendelian segregation ratio was tested using the chi-square test.

### 4.9. RNA-Seq Analysis

Two rice genotypes, Cypress and W149 mutant, were used for RNA-sequencing. Plants were grown under greenhouse conditions at the Louisiana State University Campus Greenhouse in Baton Rouge, Louisiana (30°24′41.7″ N 91°10′21.8″ W) during February–April 2018. Fully expanded leaves were collected at the booting stage (~53 days after sowing). Samples were taken from three biological replicates and were stored in a −80 °C refrigerator until RNA extraction.

Total RNA was extracted using TRIzol (Invitrogen, Carlsbad, CA, USA) based on the manufacturer’s protocol. The quality and quantity of the extracted RNA were assessed using the ND-1000 Spectrophotometer (Thermofisher Scientific, Waltham, MA, USA) and Agilent 2100 Bioanalyzer (Agilent Technologies, Santa Clara, CA, USA). DNAse treatment was done using PerfeCTa DNase I (Quantabio, Beverly, MA, USA) following the manufacturer’s instructions. Purified RNA samples were submitted to Novogene Corporation Inc. (Sacramento, CA, USA) for cDNA library construction and sequencing using the Illumina NovaSeq 6000 platform with 150-bp paired-end sequencing.

The quality of the raw reads was checked with FastQC (Babraham Bioinformatics, Cambridgeshire, UK, https://www.bioinformatics.babraham.ac.uk/), wherein reads with ≥30 Phred quality score were used in downstream analysis. The processed paired-end reads were mapped to the rice reference genome (Os-Nipponbare-Reference-IRGSP-1.0 downloaded from the Rice Genome Annotation Project) using HISAT2 version 2.1.0 [61]. The resulting mapped reads were processed using featureCounts [62] and DEGs were determined from a raw count table using “DESeq2” R package [63]. To identify the DEGs, a Benjamini–Hochberg-adjusted *p*-value of <0.05 and log2 fold change ≥1.5 were set as criteria. Gene ontology enrichment of the DEGs was performed using the singular enrichment analysis with agriGO v2 [64] to determine the biological significance with respect to biological processes (BP), molecular functions (MF), and cellular components (CC).

Transcripts of each biological sample were reconstructed to identify novel transcripts using Stringtie (v2.1.0, Johns Hopkins University, Baltimore, MD, USA; https://github.com/gpertea/stringtie) [65]. Identified transcripts were then compared with the MSU release 7 annotations [57]. The Stattest function of R package ballgown (v2. 18.0, R 3.6.2) [66] was used to test for differentially expressed transcripts between Cypress and W149 using all three biological replicates with a false discovery rate at 0.05. All raw read sequences of W149 and Cypress were deposited in the NCBI’s Sequence Read Archive (SRA) under the accession number PRJNA605404.

### 4.10. Validation of Gene Expression

To validate the gene expression patterns identified in the RNA-Seq analysis, one μg DNase-treated RNA was used to synthesize cDNA with the iScript cDNA Synthesis Kit (Bio-Rad Laboratories, Inc., Hercules, CA, USA). Representative genes were chosen from each group of different biological functions. Transcript sequences were retrieved from (RAP-DB/Phytozome) and primer pairs were designed using PrimerQuest Tool (Integrated DNA Technologies, Inc., Coralville, IA, USA) (Table S8).

Expression analyses of *Lhcb1.1* (chlorophyll a/b binding protein) (LOC_Os01g52240), indole-3-glycerol phosphate lyase (LOC_Os03g58290), photosynthetic reaction center protein (LOC_Os10g39880), *BZR4* (LOC_Os02g13900), cytochrome P450 (LOC_Os01g24780), *G1L2* (LOC_Os06g46030), and *MADS62* (LOC_Os08g38590) were performed in 96-well plates on an Applied Biosystems QuantStudio 3 Real-Time PCR system using iTaq^TM^ Universal SYBR Green Supermix (Bio-Rad Laboratories, Inc., Hercules, CA, USA) in a total reaction volume of 10 μL containing the following components: 5.0 µL of 2× iTaq^TM^ universal SYBR^®^ Green supermix, 0.3 µL of 5 µM of each primer, 1.0 µL of 1:10 cDNA, and 3.4 µL sterilized distilled water. The cycling conditions were 5 min of initial denaturation at 95 °C, followed by 40 cycles of 95 °C for 15 s, and 60 °C for 1 min. The melting curve was carried out in the 60–95 °C range. The negative control used was no-tissue control. The expression levels of the target genes and a reference gene (*EF1α*) were measured in three biological replicates per tissue using gene-specific primers. Expression levels for genes were determined using the 2^−ΔΔCT^ method as described previously [67].

### 4.11. Validation of Chimeric Nal1 Transcript

There were two types of chimeric transcripts detected from the RNA-Seq data in W149 due to the deletion of the stop codon in LOC_Os04g52479 but not in Cypress. In one transcript, the exon 3 of LOC_Os04g52479 joined to the exon 2 of the adjacent locus LOC_Os04g52500. The second transcript involved the joining of exon 4 of LOC_Os04g52479 to exon 1 of LOC_Os04g52500. To validate these chimeric transcripts, two pairs of primers were designed: Exon3-52479F (5′-CACTGGCAACAAGCAGGTTGG-3′), Exon2-52500R (5′-CATGTCGGGGTCCAGAGTGTC-3′), Exon4-52479F (5′-GGAGGCAAGTATGCAAAGTTGGC-3′), and Exon1-52500R (5′-CATCAACGGCTTCAAGAATCGAGC-3′). PCR amplification using cDNA from both W149 and Cypress was done using all combinations of forward and reverse primers following a similar PCR condition and profile described earlier. About 48 µL of each PCR sample was mixed with 2.0 µL 10× loading dye, and these were loaded in 1.5% agarose gel and electrophoresed at 120 V for 60 min. Target amplification band sizes (Cypress: No amplification and W149: 283 bp) were cut from the gel and purified using a Gel/PCR DNA fragment Extraction kit (IBI scientific, Dubuque, IA, USA). Samples were sequenced from both directions via Sanger sequencing at the Genomic Facility of the Louisiana State University.

## 5. Conclusions

Despite the important role of the *nal1* gene in plant developmental processes [36] and numerous investigations on its variants and mutants, it is still unclear how *Nal1* affects multiple traits. While most studies focused on the impacts and utility of this gene for crop improvement using natural variants [14,15,16], few studies provided a molecular basis of the physiological impacts on rice plant using mutants [13,19,23]. *Nal1* was shown to have control on some aspects of the leaf characteristics through its regulation of the cell cycle [19] and polar auxin transport [13]. Another study revealed its role in interaction with the FZP gene to increase rice yield [24]. Using a novel near-isogenic mutant, our study revealed that *nal1* does affect the whole plant architecture (not just the flag leaf) by regulating the expression of a large number of genes involved in cell proliferation and expansion, plant growth and development, reproduction, and photosynthetic apparatus. The GA responsiveness of our mutant added a new angle to our understanding of the overlapping functions of *nal1* on genes involved in hormone signaling in addition to cell cycle regulation and plant developmental processes. Along with its impact on the above processes, *nal1* was shown to regulate genes involved in GA and BR signaling pathways, contributing toward the plants’ overall architecture [68], which is in sharp contrast to earlier studies suggesting the role of *nal1* in polar auxin transport [13]. Although we demonstrated the formation of chimeric transcripts by fusion of the partial readthrough transcripts of *nal1* and the adjacent gene due to loss of the stop codon in this mutant, the role of these chimeric transcripts on plant phenotype remains to be investigated in the future.

## Figures and Tables

**Table 1:** Phenotypic characteristics of Cypress and W149 mutant. The mean value of each attribute is indicated by the mean ± standard deviation and sample size is indicated in parentheses. Asterisks indicate significant differences between Cypress and W149 mutant, as determined by Student’s *t*-test.

Attribute	Cypress	W149
Plant height (cm)	81.1 ± 1.6 (10)	50.7 ± 1.9 * (10)
Panicle length (cm)	18.6 ± 0.3 (10)	11.3 ± 0.3 * (10)
Leaf length (cm)	43.0 ± 2.0 (10)	22.9 ± 0.7 * (10)
Leaf width (cm)	1.2 ± 0.0 (10)	0.5 ± 0.0 * (10)
Leaf greenness (SPAD value)	41.9 ± 0.8 (10)	50.9 ± 0.6 * (10)
Number of large veins (LV)	9.6 ± 0.3 (5)	9.2 ± 0.4 ^ns^ (5)
Number of small veins (SV)	52.4 ± 0.7 (5)	29.6 ± 0.5 * (5)
Number of SV between LV	6.14 ± 0.3 (7)	3.6 ± 0.2 * (7)
Leaf midrib	Pronounced	Not pronounced
Leaf thickness (µm)	90.2 ± 3.3 (9)	139.1 ± 0.6 * (9)
Interveinal distance (µm)	198.1 ± 4.4 (9)	152.3 ± 4.1 * (9)
Mesophyll cell (MC) area (µm^2^)	10683.1 ± 254.5 (9)	11941.7 ± 717.6 ^ns^ (9)
Lateral MC number	10.1 ± 0.3 (9)	5.0 ± 0.1 * (9)
MC layer number	3.3 ± 0.2 (9)	4.7 ± 0.2 * (9)
Vascular area (µm^2^)	589.0 ± 29.4 (18)	963.0 ± 75.3 * (18)
Bundle sheath cell (BSC) area (µm^2^)	1557.6 ± 22.7 (18)	2759.4 ± 131.8 * (18)
Vascular bundle + BSC area (µm^2^)	2146.6 ± 31.71 (18)	3722.4 ± 190.6 * (18)
Bundle sheath cell extension number	1.9 ± 0.2 (18)	5.2 ± 0.3 *(18)
Panicle neck (mm^2^)	0.7 ± 0.0 (3)	0.2 ± 0.0 * (3)
Number of grains per panicle	270.2 ± 24.3 (9)	89.6 ± 10.0 * (9)
100-grain weight (g)	2.2 ± 0.04 (9)	1.8 ± 0.05 * (9)
Total grain weight per panicle (g)	5.9 ± 0.56 (9)	1.6 ± 0.21 * (9)

* 0.01 ≤ *p* < 0.05, ^ns^ not significant.

## Supplementary Materials

Supplementary materials can be found at https://www.mdpi.com/1422-0067/21/21/8106/s1.

## Abbreviations

GA	gibberellin
BR	brassinosteroid
QTL	quantitative trait loci
GPS	green for photosynthesis
FZP	frizzy panicle
UTR	untranslated region
LV	large vein
SV	small vein
IVD	interveinal distance
BSC	bundle sheath cells
VB	vascular bundle
MC	mesophyll cell
BSA	Bulked segregant analysis
SSR	simple sequence repeat
SNP	single nucleotide polymorphism
InDel	insertions-deletions
IBA	indole-3-butyric acid
NAA	1-Naphthaleneacetic acid
2,4-D	2,4-dichlorophenoxyacetic acid
DEG	differentially expressed genes
GO	gene ontology
qRT-PCR	quantitative real-time polymerase chain reaction
ns	nonsynonymous
ARF	auxin response factor
BP	biological process
MF	molecular function
CC	cellular component
SRA	Sequence Read Archive
