# Supplementary material for: A Novel Mutation of the NARROW LEAF 1 Gene Adversely Affects Plant Architecture in Rice (Oryza sativa L.)
Source: Int J Mol Sci. 2020 Oct 30;21(21):8106. doi: 10.3390/ijms21218106 (PMC7672626; doi:10.3390/ijms21218106)
Supplement: Supplementary file 1 [file ijms-21-08106-s001.zip › Supplementary Figures S1-S9.pdf]

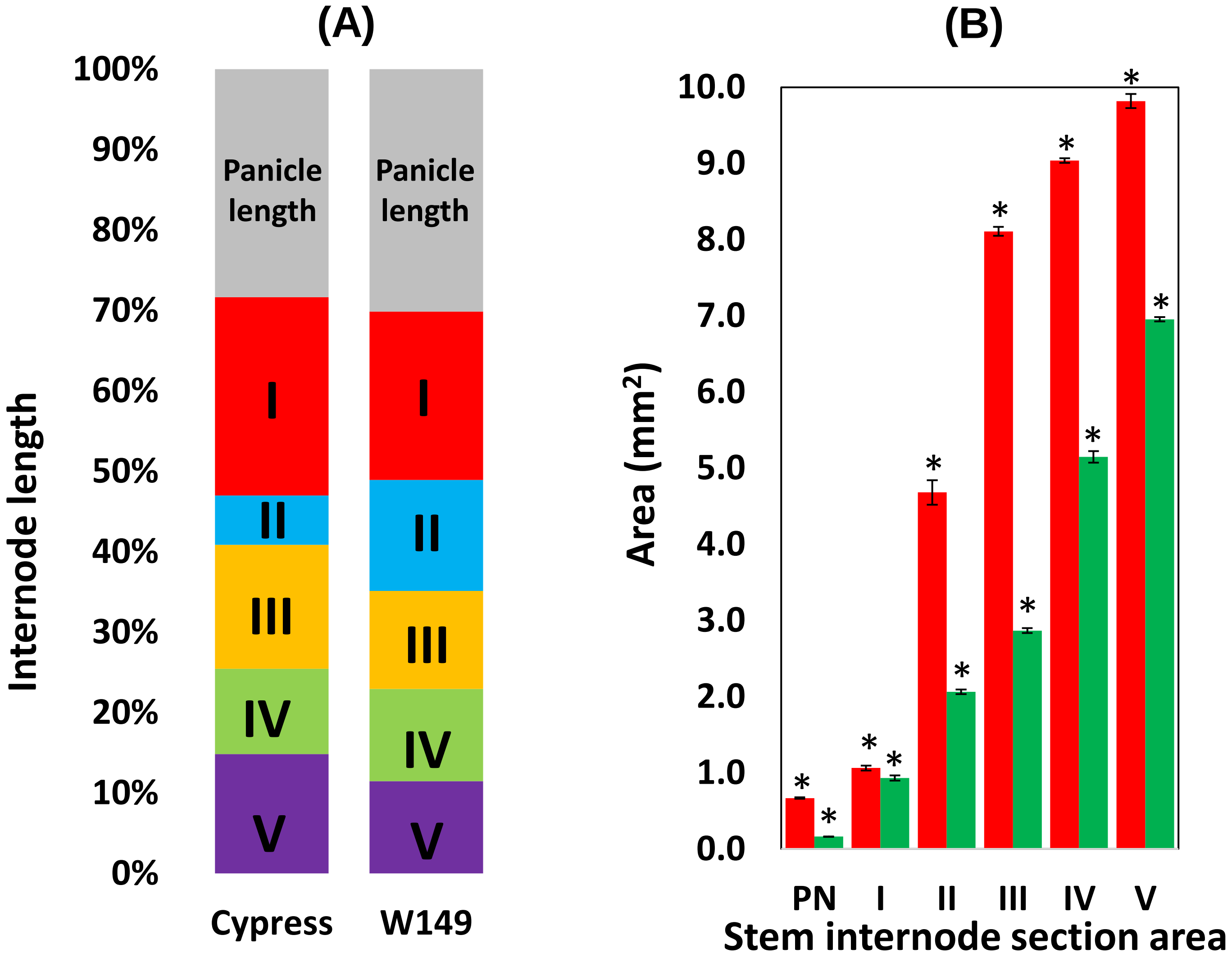

Figure S1 Comparative internode profile of Cypress and W149. (A) Schematic representation of the contribution of each internode and panicle to the plant height in Cypress (left) and W149 (right); (B) section area of each internode and panicle neck (mm^2) in Cypress (red) and W149 (green). '*' indicates significant difference between both genotype determined by Student's T-test at p=0.05.

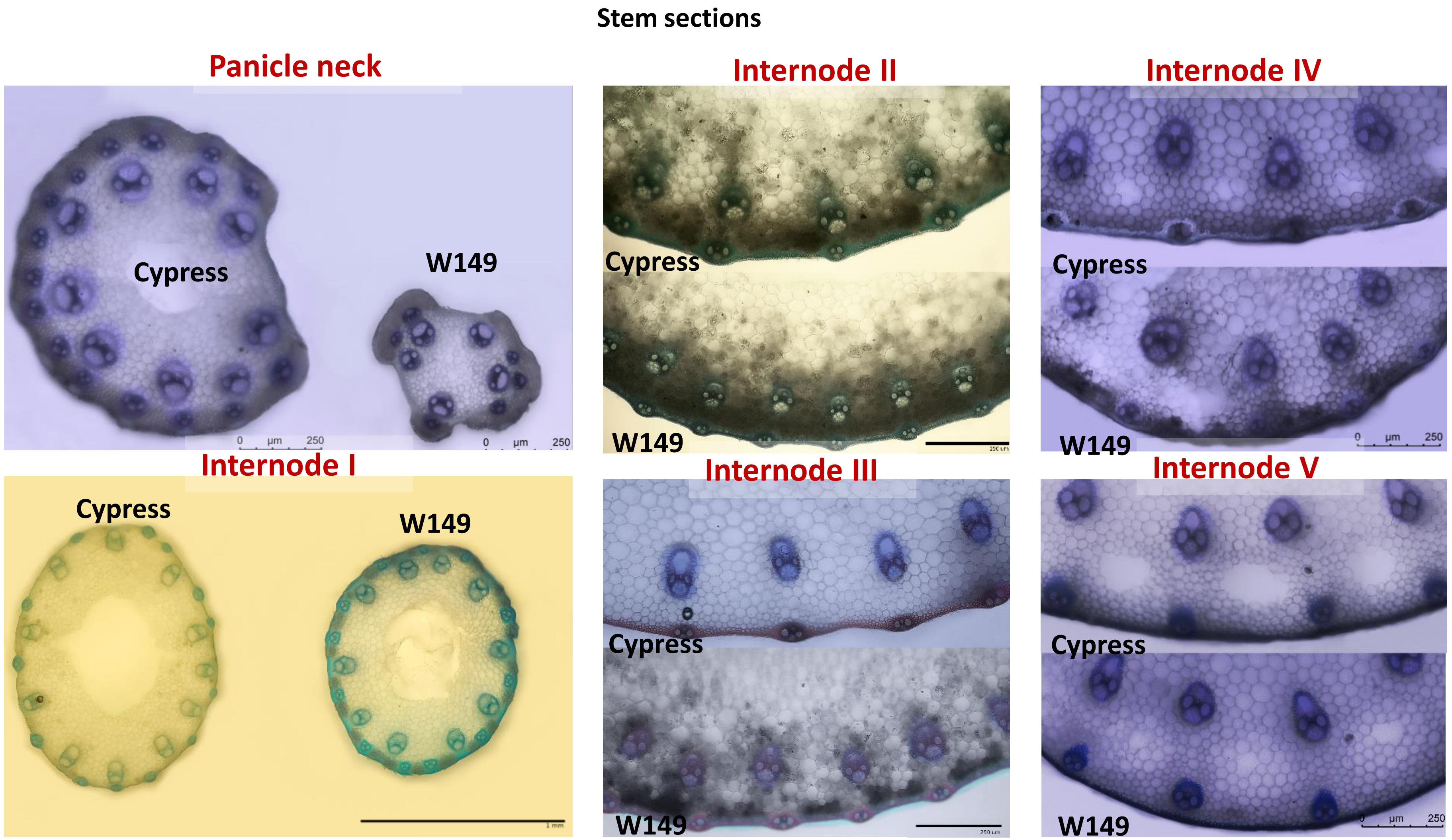

Figure S2 Stem sections of panicle neck and each internode in Cypress and W149.

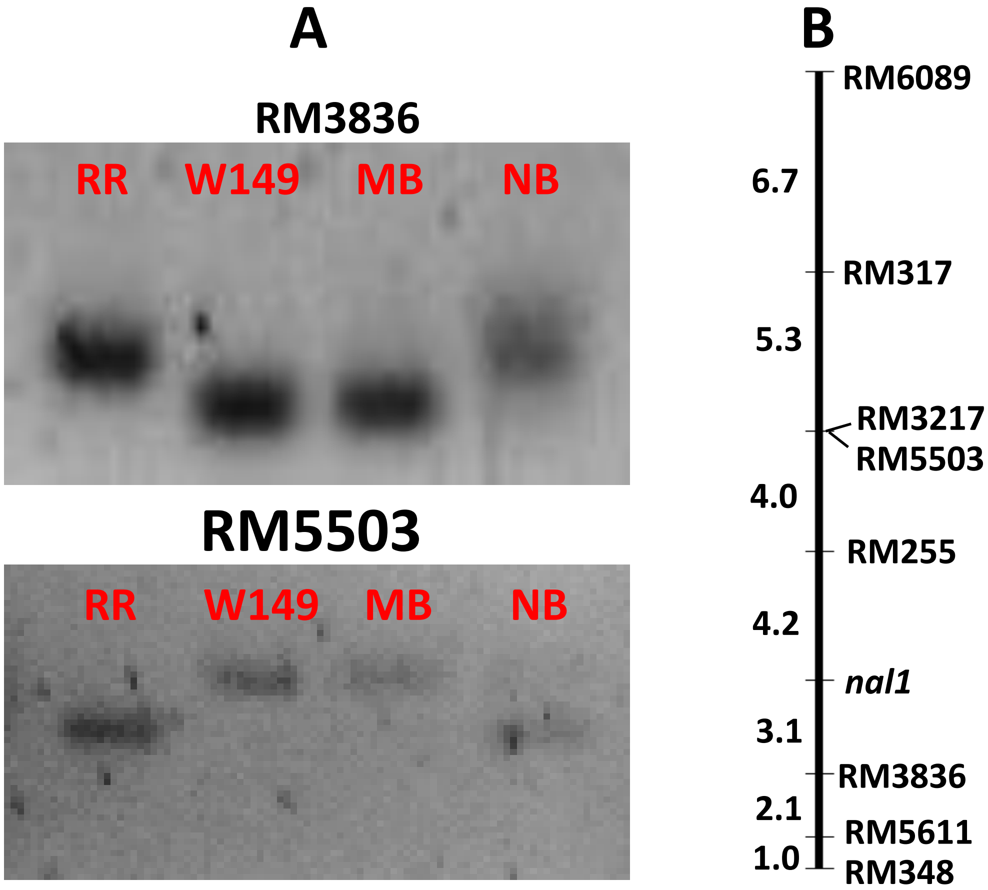

Figure S3 (A) Bulked segregant analysis using linked markers RM3836 (31.626,300 Mb) and RM5503 (30.178,482 Mb). Mutant and wild type plants were selected from the F_2 population of the cross W149 x PSRR-1 for bulked segregant analysis. RR: red rice PSRR-1, W149: weedy mutant, MB: mutant bulk, NB: normal bulk. (B) A partial linkage map of chromosome 4 showing the position of the mutant locus '*nal1*'.

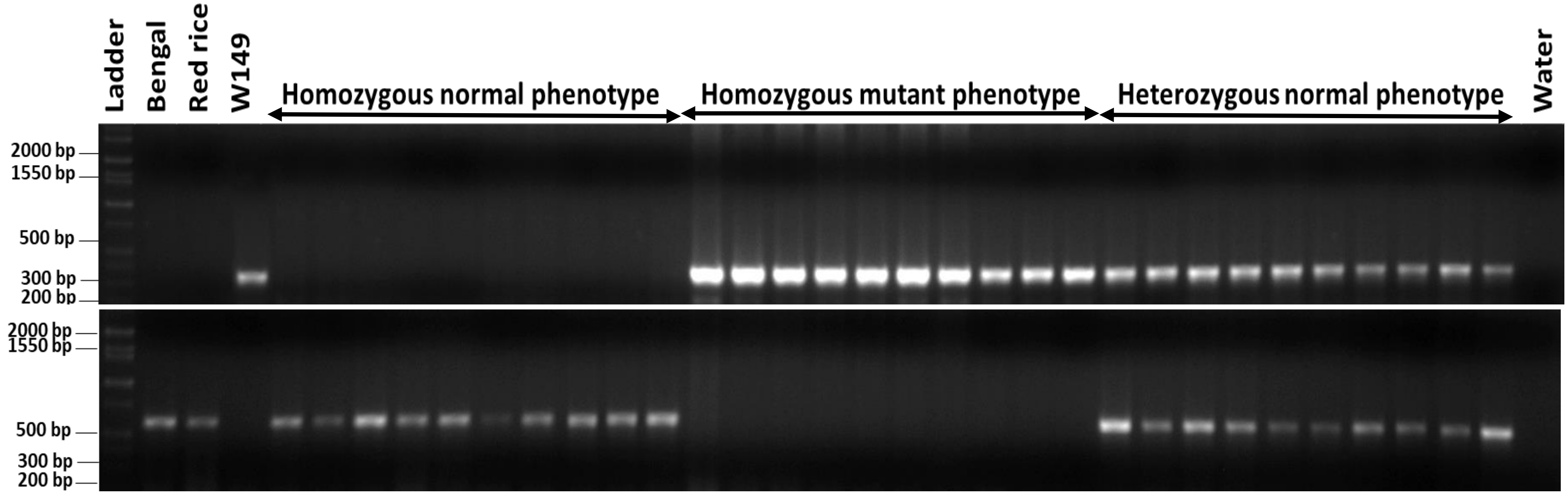

Figure S4 Genotyping of representative W149 x Bengal F_2 progenies to distinguish homozygous normal, heterozygous normal, and homozygous mutant plants. A. PCR amplification by the Primer pair 1 (1F and 1R); B. PCR amplification by the Primer pair 2 (2F and 2R).

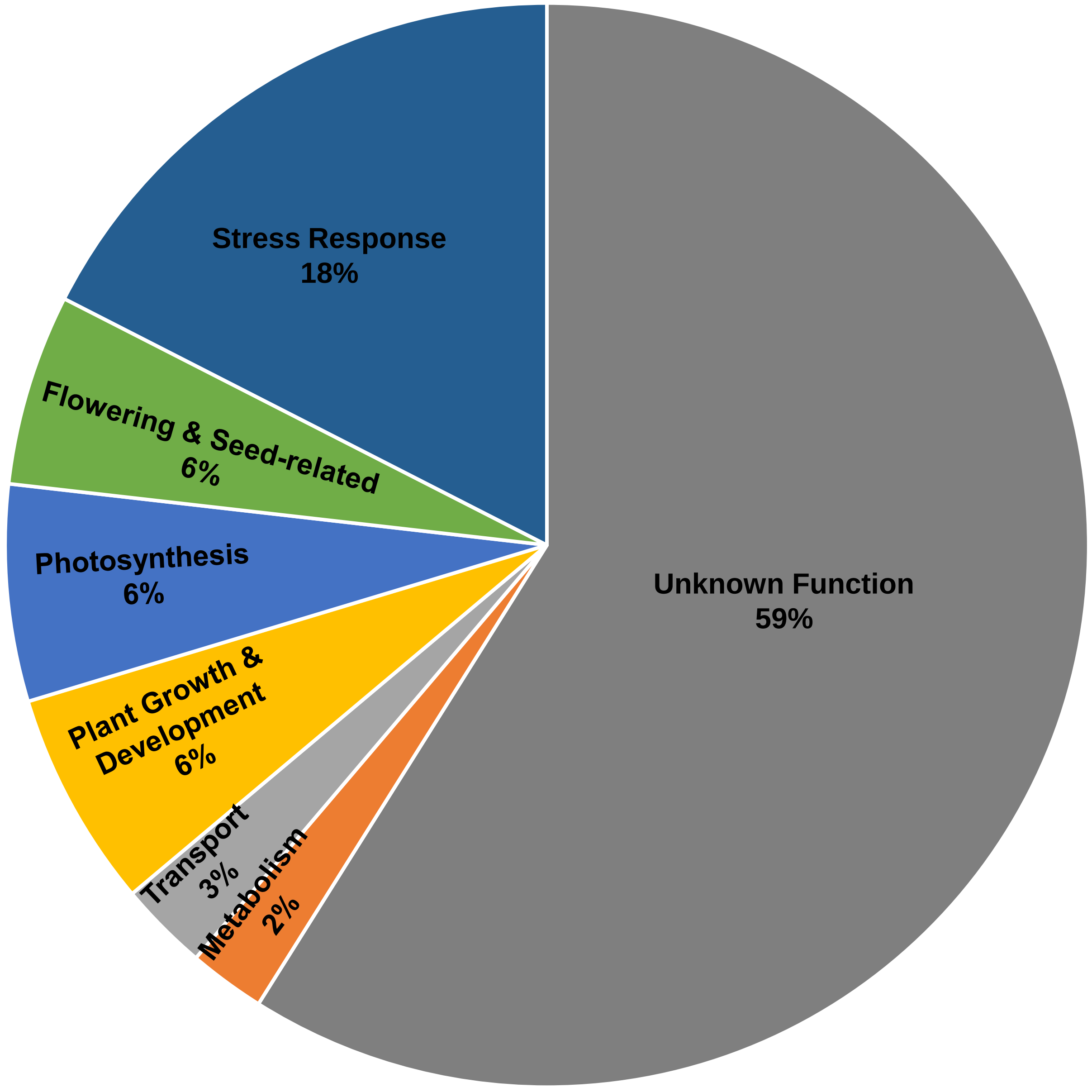

Figure S5 Clustering of 263 differentially expressed genes based on biological functions in W149 compared with Cypress.

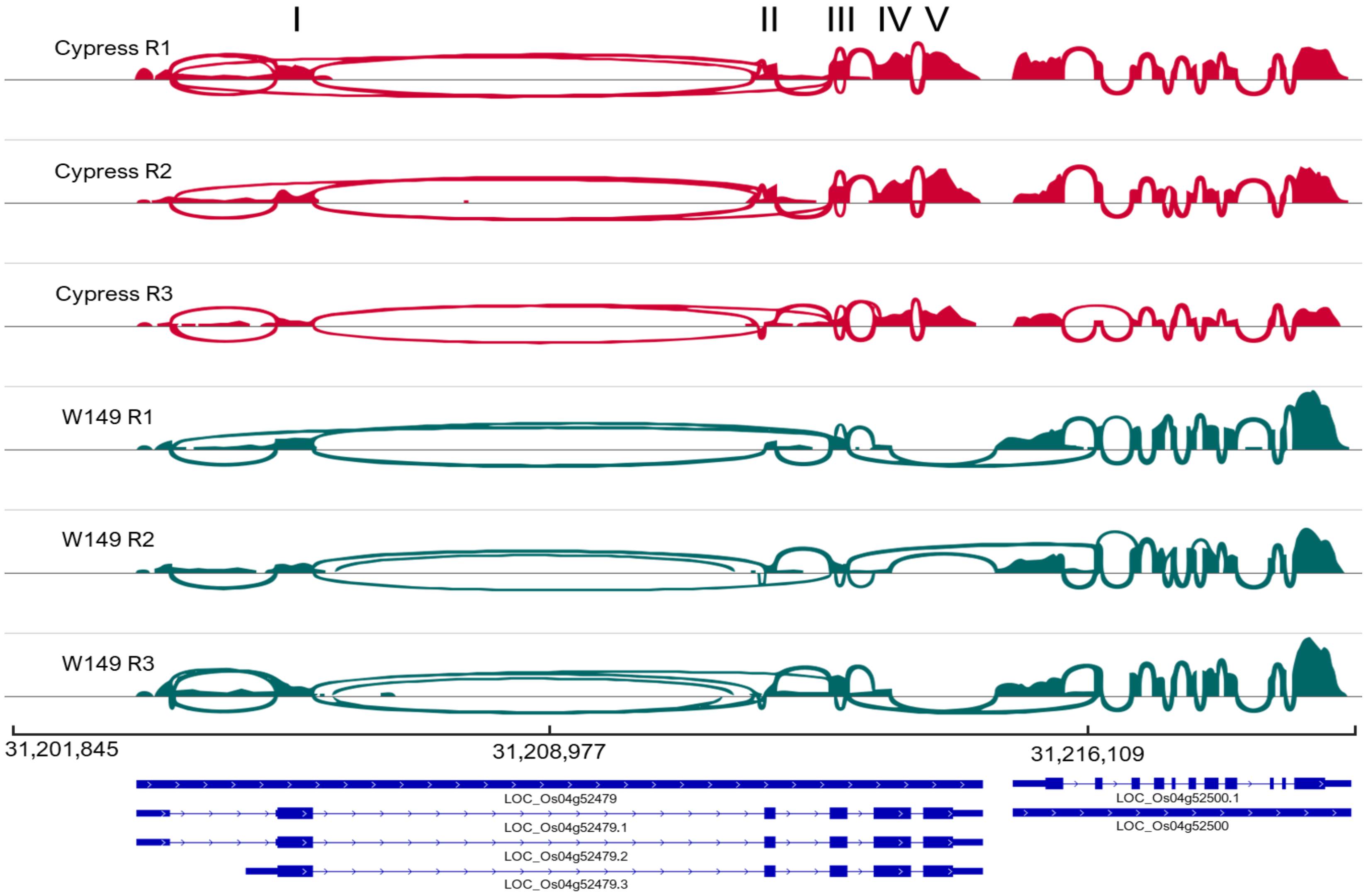

Figure S6 Sashimi plot showing RNA-seq reads mapping of *Nal1* and LOC_Os04g52500 in three biological replicates of Cypress and W149. Height of the bars represent overall read coverage. Splice junctions are displayed as loops. Exon numbers I through V are indicated on the top of the panel. MSU v7.0 gene model with three known transcript variants of *Nal1* is displayed below. Plot was visualized using IGV v.2.4.14.

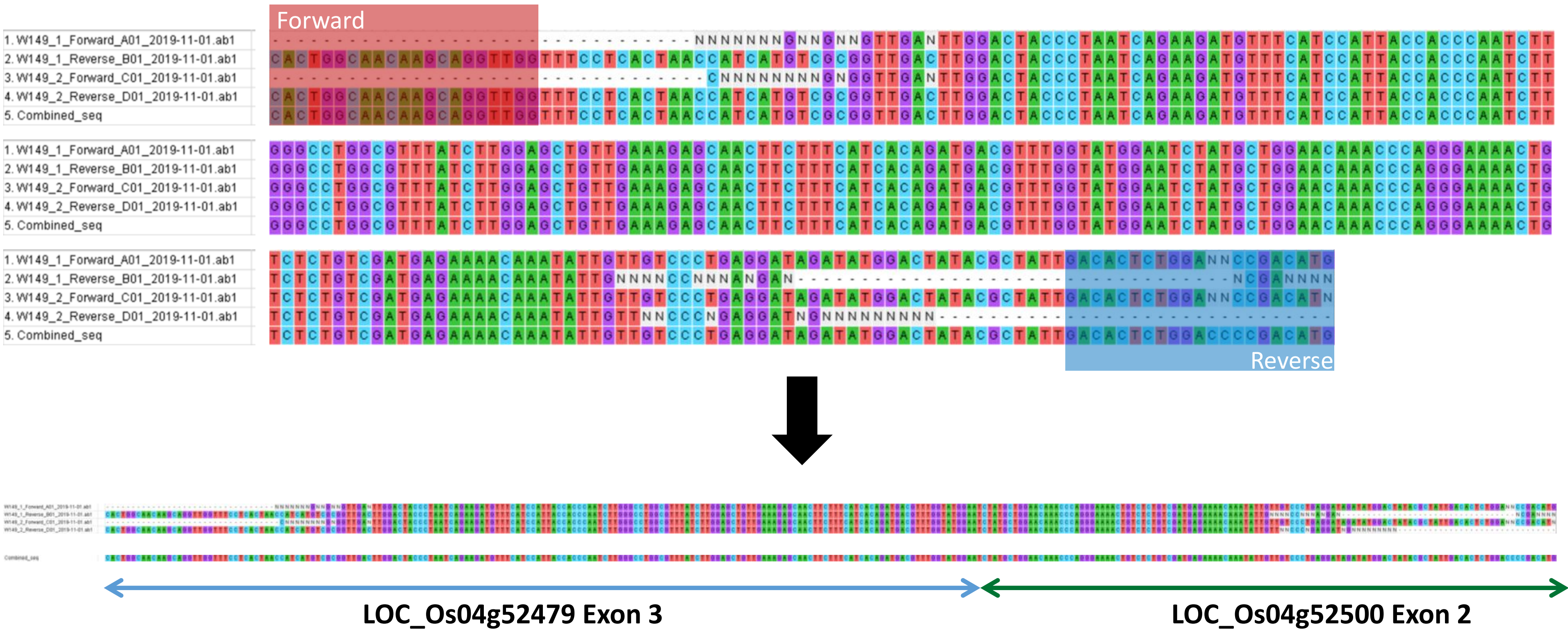

Figure S7 Confirmation of the chimeric transcript due to fusion of exon 3 of LOC_Os04g52479 merged with the exon 2 of the LOC_Os04g52500. PCR fragments flanking the junction of the exon 3 of LOC_Os04g52479 and exon 2 of the LOC_Os04g52500 was amplified and sequenced.

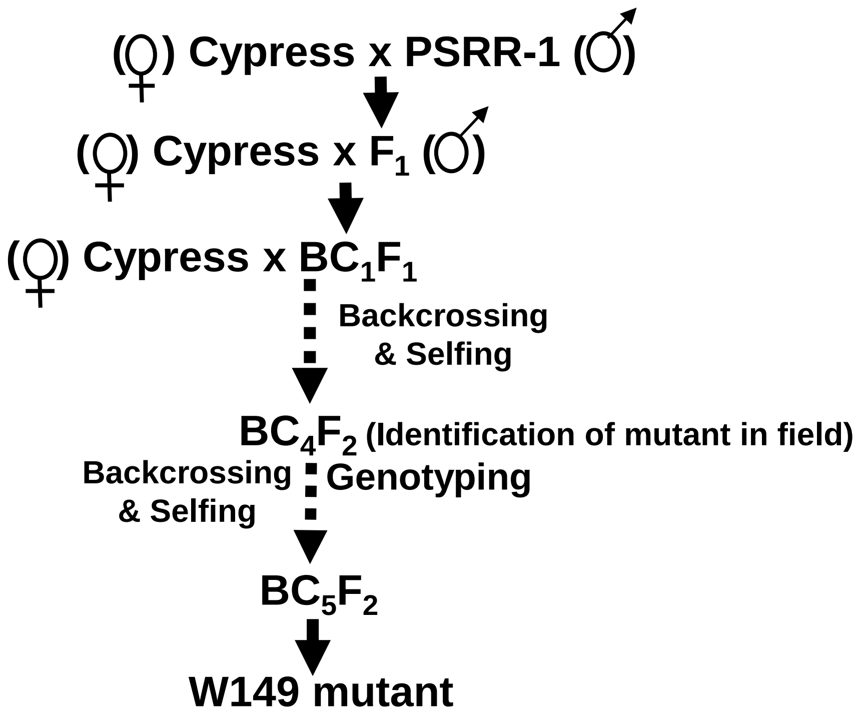

Figure S8 A schematic diagram of the origin of the narrow leaf mutant line W149 from the advanced backcross population from the cross between the cultivated rice 'Cypress' and a weedy rice accession 'PSRR-1'. Cypress was used as recurrent parent for backcrossing.

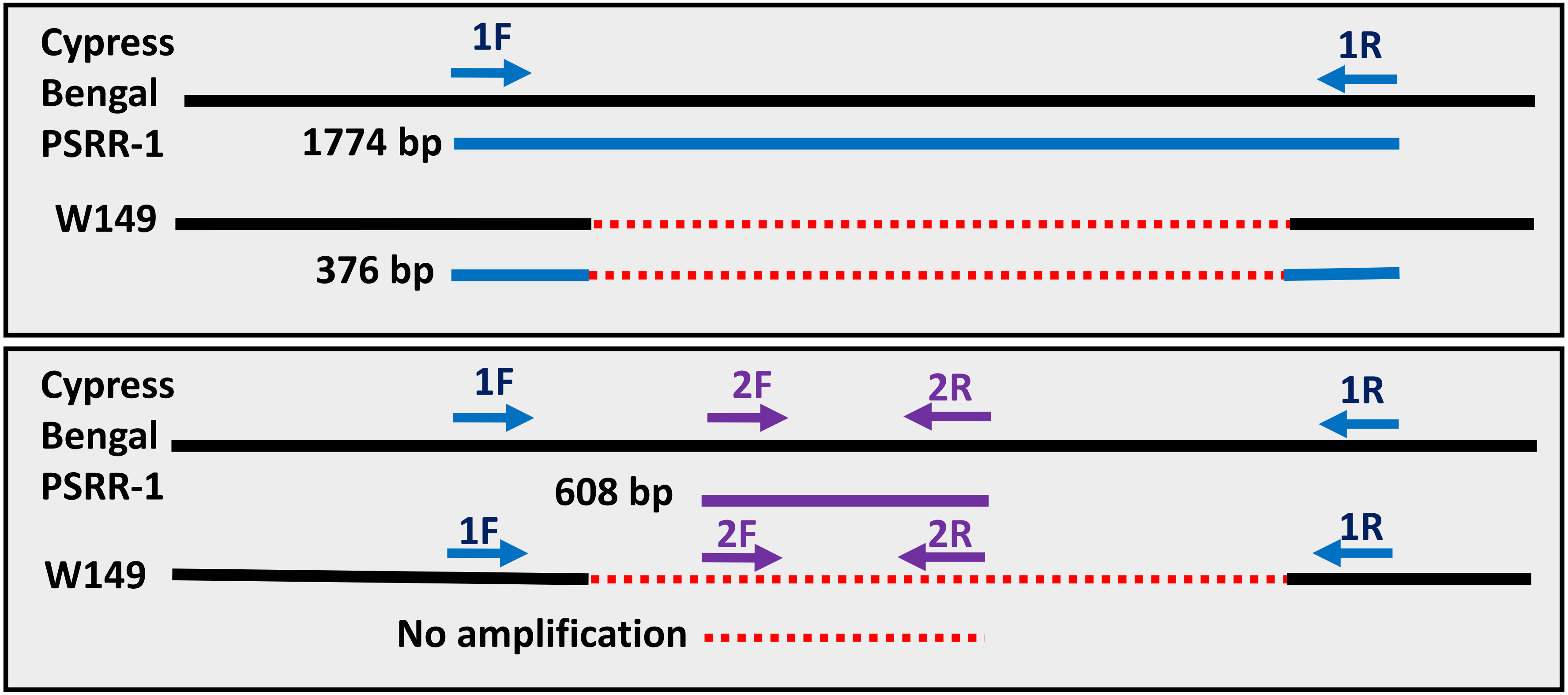

Figure S9 The principle of designing PCR primers for unambiguous genotyping of the *nal1* locus in W149, Cypress, Bengal, and PSRR-1. The PCR primers flanking the deletion showed bias in amplification particularly in heterozygous samples. Primer pair 1 (1F and 1R) spans the region of deleted region showing longer fragments (1774 bp) for Cypress, Bengal, and PSRR-1 and shorter fragment for W149 (376 bp). Primer pair 2 (2F and 2R) was used to presence and absence of the deleted region. The PCR fragment is 608 bp in Cypress, Bengal, and PSRR-1 but no amplification or fragment in W149.
