# Supplementary material for: A Novel Mutation of the NARROW LEAF 1 Gene Adversely Affects Plant Architecture in Rice (Oryza sativa L.)
Source: Int J Mol Sci. 2020 Oct 30;21(21):8106. doi: 10.3390/ijms21218106 (PMC7672626; doi:10.3390/ijms21218106)
Supplement: Supplementary file 1 [file ijms-21-08106-s001.zip › Supplementary Materials Cover page.docx]

Supplementary Materials

A novel mutation of the *NARROW LEAF 1* gene adversely affects plant architecture in rice (*Oryza sativa* L.)

Prasanta K. Subudhi, Richard S. Garcia, Sapphire Coronejo, and Teresa B. Leon

Figures included: Figure S1 to Figure S9

Tables included: Table S1 to Table S8
