# Supplementary material for: A Novel Mutation of the NARROW LEAF 1 Gene Adversely Affects Plant Architecture in Rice (Oryza sativa L.)
Source: Int J Mol Sci. 2020 Oct 30;21(21):8106. doi: 10.3390/ijms21218106 (PMC7672626; doi:10.3390/ijms21218106)
Supplement: Supplementary file 1 [file ijms-21-08106-s001.zip › Tables S1-S4 and S7-S8.docx]

**Table S1** Comparison of whole genome sequence of W149 mutant with the recurrent parent ‘Cypress’.

|  | **Total no. of variants** | **CPRS-specific variants (No.)** | **CPRS-specific variants (%)** | **PSRR-specific variants (No.)** | **PSRR-specific variants (%)** |
| --- | --- | --- | --- | --- | --- |
| Chr1 | 46012 | 44897 | 97.6 | 1115 | 2.4 |
| Chr2 | 29632 | 28719 | 96.9 | 913 | 3.1 |
| Chr3 | 19082 | 18268 | 95.7 | 814 | 4.3 |
| Chr4 | 47058 | 45873 | 97.5 | 1185 | 2.5 |
| Chr5 | 27856 | 26948 | 96.7 | 908 | 3.3 |
| Chr6 | 37882 | 37068 | 97.9 | 814 | 2.1 |
| Chr7 | 29305 | 28281 | 96.5 | 1024 | 3.5 |
| Chr8 | 30702 | 29816 | 97.1 | 886 | 2.9 |
| Chr9 | 31422 | 30851 | 98.2 | 571 | 1.8 |
| Chr10 | 38633 | 37236 | 96.4 | 1397 | 3.6 |
| Chr11 | 51363 | 49800 | 97.0 | 1563 | 3.0 |
| Chr12 | 43267 | 41862 | 96.8 | 1405 | 3.2 |
| Total/Mean | 432214 | 419619 | 97.1 | 12595 | 2.9 |

**Table S2** Phenotypic and genotypic grouping of plants in F2 population from the W149 x Cypress cross. Measurements were made on plant height, panicle length, leaf length, leaf width, and SPAD value. Based on these measurements, plants were grouped as normal or mutant category. Genotyping with *nal1* marker, plants were assigned to homozygous-normal, heterozygous-normal or homozygous-mutant categories. SE, Standard Error; Min, minimum; Max, Maximum.

|  |  | **Mean** | **SE** | **Min** | **Max** | **Sample size** |
| --- | --- | --- | --- | --- | --- | --- |
| **Genotypic grouping** |  |  |  |  |  |  |
| Cypress | Parent | 81.1 | 1.6 | 6.4 | 75.9 | 10 |
| W149 | Parent | 50.7 | 1.9 | 11.8 | 44.7 | 10 |
| Heterozygous-Normal | F_2_ | 72.4 | 0.7 | 12.4 | 63.4 | 174 |
| Homozygous-Normal | F_2_ | 78.5 | 1.0 | 12.0 | 69.1 | 83 |
| Homozygous-mutant | F_2_ | 48.1 | 0.5 | 10.4 | 43.1 | 83 |
|  |  |  |  |  | Total F_2_ | 340 |
| **Phenotypic grouping** |  |  |  |  |  |  |
| Cypress | Parent | 81.1 | 1.6 | 6.4 | 75.9 | 10 |
| W149 | Parent | 50.7 | 1.9 | 11.8 | 44.7 | 10 |
| Cypress-like F_2_ | F_2_ | 75.5 | 0.9 | 12.2 | 66.3 | 257 |
| W149 mutant-like F_2_ | F_2_ | 48.1 | 0.5 | 10.4 | 43.1 | 83 |
|  |  |  |  |  | Total F_2_ | 340 |

Phenotyping Chi square test

Mendelian segregation (3:1); X2=0.015; p value = 0.9010; df=1

Genotyping Chi square test

Mendelian segregation (1:2:1); X2=0.157; p value = 0.9245; df=2

**Table S3** Validation of the *nal1* gene segregation and its cosegegation with the mutant phenotype in F_3_ progenies of selected heterozygous F_2_ plants from the W149 x Cypress and W149 x Bengal crosses. All samples plants from F_3_ progenies were phenotyped and genotyped to confirm the association of the overall mutant phenotype with *nal1* mutation.

| **Cross and selected heterozygous F_2_ plant** | **Homozygous**  **Wild type** | **Homozygous**  **Mutant type** | **Heterozygous Wild type** | **Total number of plants sampled** |
| --- | --- | --- | --- | --- |
| W149 x Cypress - 17 | 7 | 1 | 10 | 18 |
| W149 x Cypress - 69 | 6 | 6 | 6 | 18 |
| W149 x Cypress - 136 | 5 | 5 | 10 | 20 |
| W149 x Cypress – 235 | 6 | 4 | 7 | 17 |
| W149 x Bengal - 20 | 6 | 4 | 5 | 15 |
| W149 x Bengal - 25 | 3 | 2 | 15 | 20 |

**Table S4** Summary of Illumina RNA-Sequencing reads in three biological replicates of W149 and Cypress.

|  | Assembled  Read Count | Unassembled  Read Count | Total Number of Sequences | Assembly  Rate (%) |
| --- | --- | --- | --- | --- |
| Cypress_R1 | 46331999 | 2033437 | 48359668 | 95.8 |
| Cypress_R2 | 54872904 | 2630324 | 57501974 | 95.4 |
| Cypress_R3 | 44477413 | 2158311 | 46632634 | 95.4 |
| W149_R1 | 54424553 | 2401653 | 56816472 | 95.8 |
| W149_R2 | 47108204 | 2085260 | 49190514 | 95.8 |
| W149_R3 | 51585916 | 2041752 | 53620778 | 96.2 |

**Table S7** Identified transcripts and their expression level (FPKM values) in Cypress and W149 using Stringtie.

| **Transcript name** | **Start** | **End** | **Transcript length (bp)** | **Mean FPKM^*^ (Cypress)** | **Mean FPKM (W149)** |
| --- | --- | --- | --- | --- | --- |
| LOC_Os04g52479.1 | 31203525 | 31214741 | 2616 | 1.294 | 0.000 |
| LOC_Os04g52479.2 | 31203525 | 31214741 | 2621 | 4.578 | 0.000 |
| LOC_Os04g52479.3 | 31204979 | 31214741 | 2565 | 0.912 | 0.000 |
| LOC_Os04g52500.1 | 31215136 | 31219715 | 2479 | 10.334 | 1.018 |
| Nal1-Cht2 | 31203504 | 31219715 | 3160 | 0.000 | 3.105 |
| Nal1-Cht1 | 31203505 | 31219715 | 4271 | 0.000 | 3.142 |

**^*^**FPKM, fragments per kilobase of transcript per million reads (FPKM)

**Table S8** Primers for qRT-PCR analysis

| **Gene Name (Locus ID)** | **Forward primer** | **Reverse primer** |
| --- | --- | --- |
| *Lhcb1.1* (LOC_Os01g52240) | GTCAACAACAACGCATGG | CAATCTGAACGAACGAAGC |
| *Indole-3-glycerol Phosphate Lyase* (LOC_Os03g58290) | CAGCTTCTCCCAAACAAGG | CGTTTATTTGTCCATCGTTTCC |
| *Photosynthetic Reaction Center Protein* (LOC_Os10g39880) | CCTACTTCTACGGCGATTGGATTG | TACACCATCAAAGAAACTTCCTTGAC |
| *BZR4* (LOC_Os02g13900) | TCCTCCTCCCAGATCACG | GCCGAAGTAGGAGTAGTGC |
| *Cytochrome P450* (LOC_Os01g24780) | AGGGTCCAAGAGGTTGCATCG | GCACGAACCGTAGAGATCGACAC |
| *G1L2* (LOC_Os06g46030) | CTCCGAGCTAAGCCACTCCAC | CCTAGCACAAGCAGCAGCAAAC |
| *MADS62* (LOC_Os08g38590) | CAGTACCTGTCGATGGAGCATG | GTAGGTTAGTTAGGTGAGGTCGATC |
